# Osteoradionecrosis in patients with head and neck cancer after reirradiation – a systematic review and meta-analysis

**DOI:** 10.1016/j.ctro.2026.101342

**Published:** 2026-10-28

**Authors:** Annemarie Fritz, Nils Gleim, Anita Kloss-Brandstätter, Thomas Kuhnt, Nils H. Nicolay, Andreas Maximilian Fichter, Sebastian Gaus, Alexander Rühle

**Affiliations:** a Department of Oral and Maxillofacial Surgery, University of Leipzig Medical Center, Leipzig, Germany; b Comprehensive Cancer Center Central Germany, Partner Site Leipzig, Leipzig, Germany; c Department of Radiation Oncology, University of Leipzig Medical Center, Leipzig, Germany; d Department of Engineering & IT, Carinthia University of Applied Sciences, Villach, Austria; e Department of Radiation Oncology, Princess Margaret Cancer Centre, University of Toronto, Toronto, Canada

**Keywords:** Head and neck cancer, radiotherapy, reirradiation, recurrence, chemotherapy, mandible, maxilla

## Abstract

**Objective:**

To determine the observed incidence and risk factors of osteoradionecrosis (ORN) following reirradiation for recurrent or second primary head and neck cancer.

**Methods:**

A systematic review with meta-analysis was conducted according to the PRISMA guidelines, with the study protocol prospectively registered with PROSPERO (CRD42025635226). A literature search was performed in PubMed/MEDLINE and Web of Science. Randomized and observational studies investigating reirradiation for recurrent or second primary head and neck tumors were included. The primary endpoint was occurrence of ORN of the maxilla and/or mandible.

**Results:**

Of 580 identified records, 62 studies involving 3278 patients (median age, 61 years; 2087 [63.7%] male) were included, among them 14 prospective and 48 retrospective studies. Teletherapy was administered in 2546 patients (77.7%), and brachytherapy in 684 patients (20.9%). At a median follow-up of 26 months, the pooled observed incidence of ORN of the mandible and/or maxilla was 3.6% (95% CI, 2.8%–4.6%). When restricted to patients evaluable for late adverse events, pooled observed ORN incidence was 4.0% (95% CI, 3.0%–5.3%). A higher study-level proportion of patients receiving adjuvant reirradiation was associated with increased observed ORN incidence (β = 1.091; 95% CI, 0.256–1.926; *P* = .01), whereas radiation dose, modality, and interval between primary radiotherapy and reirradiation were not predictive.

**Conclusion:**

This meta-analysis demonstrates that the observed incidence of ORN after reirradiation for recurrent or second primary head and neck cancer is approximately 4%. These findings provide a benchmark for patient counseling, while highlighting the need for standardized ORN assessment in future studies.

## Highlights

• First meta-analysis of ORN incidence after reirradiation for head and neck cancer.
• Analysis included 62 studies with 3278 patients treated with reirradiation.
• Observed incidence of ORN of the mandible and/or maxilla was 3.6% (95% CI, 2.8–4.6%).
• Pooled ORN incidence including non-jaw osseous sites was 4.7% (95% CI, 3.6%–6.1%).
• Grade 4 ORN represented the most frequently reported severity level.

## 1 Introduction

Head and neck cancer (HNC) is the seventh most common cancer worldwide, with approximately 950,000 new cases per year [1]. A significant proportion of patients develop locoregional recurrence or second primary tumors after initial treatment, posing a major therapeutic challenge due to prior treatments and limited salvage options [2], [3].

Reirradiation has emerged as a potentially curative or palliative treatment modality for selected patients with recurrent or second primary HNC [4]. Advances in radiation techniques, including intensity-modulated radiotherapy (IMRT) and stereotactic body radiation therapy (SBRT), have improved dose conformity and normal tissue sparing [5], [6]. Nevertheless, reirradiation remains associated with a considerable risk of severe late adverse events due to cumulative dose exposure in previously irradiated tissues [7].

Among these, osteoradionecrosis (ORN) remains one of the most challenging complications associated with reirradiation for HNC. This late-onset condition profoundly affects the quality of life of cancer patients and survivors, often persisting for long times after treatment [8], [9]. Clinically, ORN is characterized by exposed or necrotic bone, which may become superinfected, and is often accompanied by symptoms such as oral or extraoral fistulas, pain, trismus, facial numbness, and deformity [10]. The underlying pathophysiology of ORN is primarily attributed to radiation-induced vascular damage, which leads to reduced blood supply and subsequent bone necrosis. The mandible is particularly susceptible due to its relatively poor vascularity [11].

While the incidence of ORN after primary radiotherapy has been widely reported, estimates vary considerably, typically ranging between 2% and 15%, reflecting heterogeneity in patient populations, treatment techniques, follow-up duration and diagnostic criteria [12]. Established risk factors include high radiation dose, surgical interventions involving the mandible, inadequate dental clearance before or dental interventions after radiotherapy, and patient-related factors such as tobacco and alcohol use [13], [14], [15], [16], [17].

In the setting of reirradiation, the risk of ORN is hypothesized to be higher due to cumulative dose effects and compromised tissue vascularity. However, reported incidence rates remain inconsistent, and the true cumulative incidence of ORN in this population is unknown [12]. Therefore, the aim of this systematic review and meta-analysis was to determine the observed incidence of ORN in patients with recurrent or second primary HNC undergoing reirradiation. In addition, we sought to identify potential risk factors associated with the development of ORN to better inform clinical decision-making and patient counseling.

## 2 Methods

### 2.1 Study design

This systematic review was conducted according to the 2020 Preferred Reporting Items for Systematic Reviews and Meta-Analyses (PRISMA) guidelines [18], [19]. A prospective analysis protocol was developed and registered in the PROSPERO database (CRD42025635226). The final protocol is available in the Supplementary Material A. The Ethics Committee of the University of Leipzig provided a waiver for this study on February 20, 2025.

A systematic literature search was performed in PubMed/MEDLINE and Web of Science between February 1, 2025, and March 31, 2025. The full search strategies for each database are provided in the Supplementary Material B. Reference management was performed using Citavi version 6.0 (Lumivero, Denver, CO, USA). Two reviewers (AF and SG) independently screened titles and abstract in duplicate. Reference lists of relevant reviews and guidelines were manually screened to identify additional eligible studies. Potential overlap between study cohorts was assessed, and in case of overlapping populations, only the largest cohort had also provided extractable data on ORN was included. Publications were considered overlapping if they originated from the same institution, study group or registry and covered overlapping treatment periods; earlier reports, interim analyses and updates of the same cohort were excluded. Publications based on registry or multi-institutional data were identified through the systematic database search and assessed like all other publications. Study selection, data extraction and risk of bias (RoB) assessment were performed independently by two reviewers (AF and SG). Discrepancies were resolved through discussion and re-evaluation of the data; if consensus could not be reached, a third reviewer (AR) was consulted. Three methodological amendments were made to the registered PROSPERO protocol before study selection: restricting the search to studies from 1995 onwards, refining case-series eligibility to consecutively included patients, and specifying the target population as recurrent or second primary HNC.

### 2.2 Selection criteria

The CoCoPop framework (Condition, Context, Population) was used to guide the eligibility criteria [20]. Studies were required to address ORN of the maxilla and mandible (condition) in the setting of reirradiation (context) in adult patients (≥18 years) with locally or locoregionally recurrent or second primary HNC (population). ORN was defined as treatment-related adverse event following reirradiation, as reported in the included studies and graded according to the Common Terminology Criteria for Adverse Events (CTCAE) or comparable classification systems. ORN was considered a late adverse event and was therefore only included if occurring ≥90 days after completion of reirradiation. When the exact time of ORN occurrence was not reported, studies were considered eligible if ORN was explicitly reported as a late adverse event or within the late toxicity assessment. Only studies that explicitly reported the occurrence or absence of ORN were included.

Reirradiation was defined as a subsequent course of teletherapy or brachytherapy administered after a prior completed course of radiotherapy, with or without neoadjuvant, concurrent, or adjuvant systemic anticancer therapy, and with or without prior surgical resection (Supplementary Material C). Both type 1 reirradiation (overlapping treatment volumes) and type 2 reirradiation (non-overlapping treatment volumes) were considered for this analysis [21]. Only randomized controlled trials and cohort studies were included, whereas case series were considered eligible only when patients were consecutively included.

### 2.3 Data extraction

Data extraction was performed using a standardized template in Microsoft Excel version 2025 (Microsoft Corporation, Redmond, WA, USA). Detailed data on all extracted variables are provided in the Supplementary Material D. For studies in which information on ORN was missing, or where ORN was reported but not precisely localized or graded, the corresponding author was contacted for clarification. In cases where the number of patients evaluated for late adverse events was not explicitly reported, the data were estimated from Kaplan-Meier curves using a predefined method described in the Supplementary Material E.

### 2.4 RoB assessment

The methodological quality of the included studies was independently assessed by two reviewers (AF and SG) (Supplementary Material F). For non-comparative and comparative retrospective studies, the MINORS (Methodological Index for Non-Randomized Studies) tool was used to systematically evaluate study design and data collection [22]. For the one randomized controlled trial analyzed in this meta-analysis, the Cochrane Risk of Bias 2 (RoB 2) tool was applied [23]. For the primary outcome of ORN occurrence, an adapted version of the JBI Critical Appraisal Checklist for Studies Reporting Prevalence Data was employed, as ORN was reported in the included studies only as a secondary outcome [20]. This assessment was also performed independently by two reviewers (AF and SG). This combined approach was intended to provide a comprehensive assessment of both the overall study methodology and reliability of the reported ORN data.

### 2.5 Statistical analysis

Statistical analyses and meta-analyses were performed using R statistical software (R Foundation for Statistical Computing, Vienna, Austria). Details of the statistical analysis are provided in the Supplementary Methods.

## 3 Results

### 3.1 Characterization of the included studies

The initial systematic search identified 580 records before removal of duplicates. A substantial number of studies addressed reirradiation and treatment-related adverse events but did not provide sufficient data on ORN, particularly regarding its occurrence or localization (Supplementary Material G).

A total of 62 studies were included in the final analysis, of which 14 were prospective and 48 retrospective in design (Table 1). Further details on the study selection process and study characteristics are provided in the PRISMA flow diagram (Fig. 1). The included studies reported on 3278 patients.

**Table 1:** Study design, sample size, patient characteristics, radiation details, and follow-up of included studies.

Author	Design	n (reirradiation)	Radiation dose reirradiation, median (Gy)	Initial radiation dose, median (Gy)
Al-Wassia et al. 2015	retrospective cohort study, non-comparative	27	60	
Awan et al. 2018	prospective non-randomized phase II single-arm trial	45	60	70
Bhalavat et al. 2018	retrospective cohort study, non-comparative	14		66
Bahl et al. 2018	retrospective cohort study, non-comparative	20	43.65	59.75
Beddok et al. 2023	retrospective cohort study, non-comparative	23		70
Benchalal et al. 1995	prospective non-randomized single-arm pilot study	19	60	63.6
Berger et al. 2010	Prospective phase II single-arm trial	57	40	64
Bots et al. 2017	retrospective cohort study, non-comparative	137	60	68
Boustani et al. 2021	retrospective cohort study, non-comparative	33	66	69.6
Buglione et al. 2015	retrospective cohort study, non-comparative	75		
Bussu et al. 2024	retrospective cohort study, non-comparative	34	30	
Cengiz et al. 2011	retrospective cohort study, non-comparative	46	30	61
Chen et al. 2007	retrospective cohort study, non-comparative	113		64
Chen et al. 2011	retrospective cohort study, non-comparative	21		66
Crevoisier et al. 2001	prospective non-randomized single-arm pilot study	25	60	55
Cvek et al. 2016	retrospective cohort study, non-comparative	40	48	
Dionisi et al. 2019	retrospective cohort study, non-comparative	17	60	70
Dornoff et al. 2015	retrospective cohort study, comparative	66	50.4	64
Duprez et al. 2009	retrospective cohort study, non-comparative, observational	84	69	61
Embring et al. 2021	retrospective cohort study, non-comparative, observational	54	59	68
Hall et al. 2003	prospective cohort study, non-comparative, observational	18	60	
Hegde et al. 2018	retrospective cohort study, non-comparative, observational	20	36.5	
Hepel et al. 2005	retrospective cohort study, non-comparative, observational	30	34	59
Held et al. 2019	retrospective cohort study, non-comparative, observational	229	51	67.4
Heron et al. 2009	prospective non-randomized phase I single-arm trial	25		64.7
Hsieh et al. 2024	retrospective cohort study, non-comparative, observational	22	60	68
Hoell et al. 2023	retrospective cohort study, non-comparative, observational	45	45	70.2
Hwang et al. 1998	retrospective cohort study, non-comparative, observational	64	60	65
Jiang et al. 2019	retrospective cohort study, non-comparative, observational	61		60
Jensen et al. 2010	retrospective cohort study, non-comparative, observational	21	45	66
Kakria et al. 2014	retrospective cohort study, non-comparative, observational	31	60	70
Karam et al. 2016	retrospective cohort study, non-comparative, observational	42		
Kramer et al. 2005	prospective non-randomized Phase I/II single-arm trial	38	42	64.2
Lee et al. 2017	prospective, non-randomized cohort study, comparative	20		
Lee et al. 2016	retrospective cohort study, non-comparative, observational	66	70	
Low et al. 2006	retrospective cohort study, non-comparative, observational	36		
Megahed et al. 2024	retrospective case series, non-comparative, observational	9	60	70
Martínez-Fernández et al. 2017	prospective non-randomized phase I/II single-arm trial	63	40	
Orlandi et al. 2019	retrospective cohort study, non-comparative, observational	159	66	
Ozyigit et al. 2011	retrospective cohort study, comparative, observational	27	57	67.4
Pialat et al. 2021	retrospective cohort study, non-comparative, observational	25	57	62
Ritter et al. 2016	retrospective, matched cohort study, observational	15		68.1
Roh et al. 2009	retrospective cohort study, non-comparative, observational	36	30	70.2
Romesser et al. 2016	retrospective cohort study, non-comparative, observational	92	60.6	61.4
Rühle et al. 2020	retrospective cohort study, non-comparative, observational	48	58.4	68
Rudzianskas et al. 2014	randomized controlled trial	63		66
Schaefer et al. 2000	prospective non-randomized Phase II single-arm trial	32		60
Salama et al. 2006	retrospective cohort study, non-comparative, observational	115	64.8	67.5
Seiwert et al. 2008	prospective non-randomized phase I single-arm trial	29	70	63
Siddiqui et al. 2009	retrospective cohort study, non-comparative, observational	21		63.5
Spencer et al. 2007	Prospective non-randomized phase II single-arm trial	79		65.2
Suh et al. 2008	retrospective cohort study, non-comparative, observational	12		63
Sulman et al. 2009	retrospective cohort study, non-comparative, observational	74	60	60
Takiar et al. 2016	retrospective cohort study, non-comparative, observational	206		
Teo et al. 1998	retrospective cohort study, non-comparative, observational	117	66	
Tselis et al. 2011	retrospective cohort study, non-comparative, observational	74		60
Uysal et al. 2023	retrospective cohort study, non-comparative, observational	10	26.25	
Vischioni et al. 2020	prospective non-randomized phase II single-arm trial	51	60	60
Voynov et al. 2006	retrospective cohort study, non-comparative, observational	22	22.5	
Waldron et al. 2019	retrospective cohort study, non-comparative, observational	26	60	60
Zheng et al. 2004	retrospective cohort study, comparative, observational	117		70
Zwicker et al. 2011	retrospective cohort study, non-comparative, observational	38	49	61

**Fig. 1:**
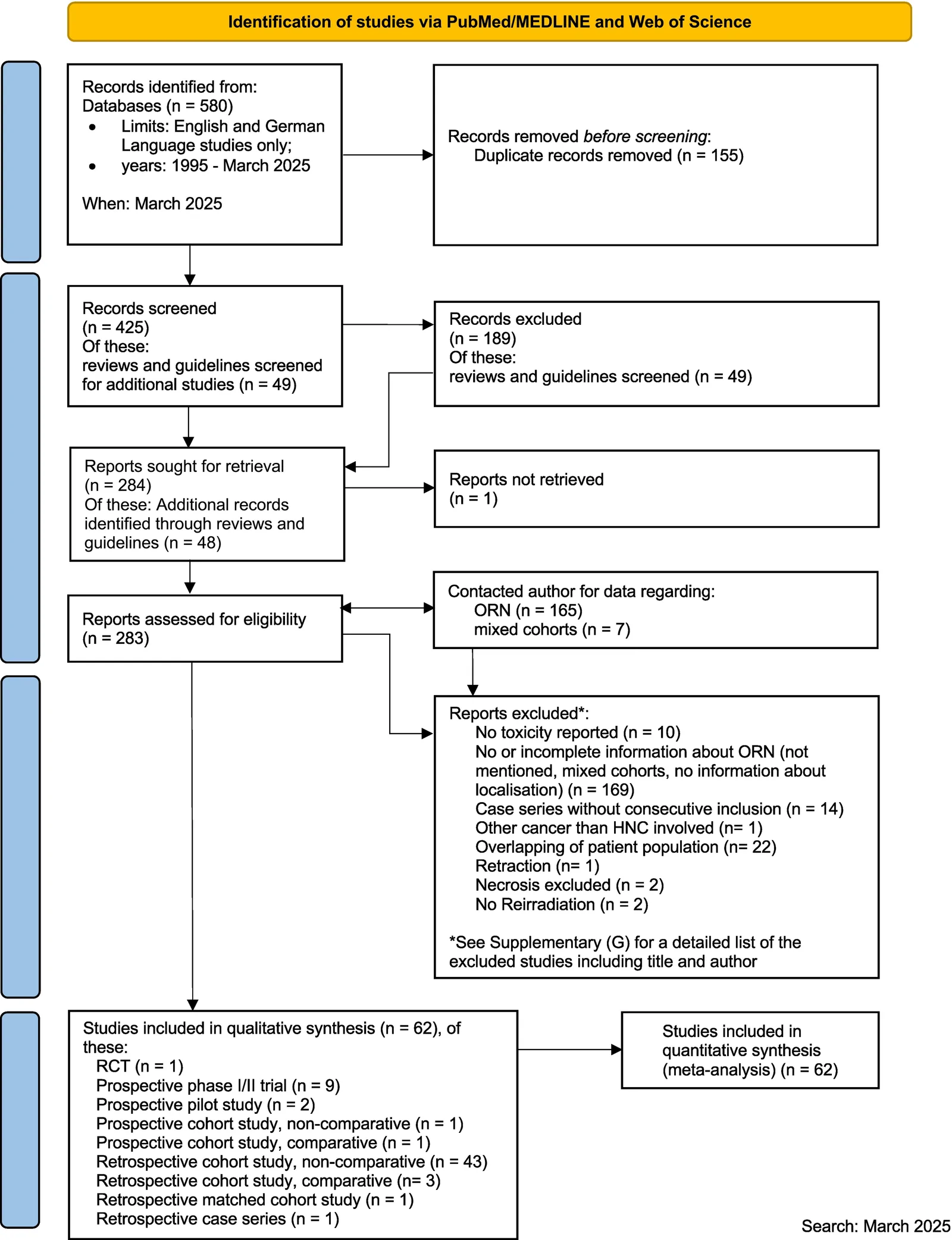
PRISMA flow diagram of study selection.

### 3.2 RoB assessment

The methodological quality of the included studies was generally moderate, with considerable variability across studies (Supplementary Material H). Based on the MINORS assessment, most non-comparative studies achieved moderate scores, with common limitations including retrospective design, lack of prospective data collection, and incomplete reporting of endpoints and follow-up. Comparative studies showed similar limitations, particularly regarding baseline comparability and outcome assessment. The adapted JBI checklist for the outcome “occurrence of ORN” revealed additional limitations in outcome reporting. In many studies, ORN was not the primary endpoint, and information on its occurrence, localization, and grading was frequently incomplete or inconsistently reported. Overall, these findings indicate a moderate RoB, particularly related to study design and outcome reporting.

### 3.3 Patients and treatment characteristics

Across all studies, the pooled median for patient age was 61 years (range, 17–97 years), with a predominance of male patients (*n* = 2087) compared to female patients (*n* = 762) among patients with reported sex (Table 1, Supplementary Material D). Although the predefined eligibility criteria restricted inclusion to adult patients (≥18 years), a small number of included studies reported age ranges extending below 18 years. As these studies predominantly enrolled adult populations and individual patient data were not available, they were retained in the analysis. The most common histology was squamous cell carcinoma (*n* = 1755). The nasopharynx was the most frequently involved site of recurrence or second primary tumor (*n* = 669), and the majority of cases represented recurrent disease (*n* = 2684).

Regarding treatment characteristics, 1049 patients received adjuvant reirradiation, whereas 2156 patients underwent definitive reirradiation (Table 2). Treatment intent was not reported in all included studies; therefore, these numbers do not account for the entire study population. Systemic therapy, including chemotherapy or targeted therapy, was administered in 1226 patients.

**Table 2:** Details about type of reirradiation and osteoradionecrosis (ORN) characteristics of included studies.

Author	Reirradiation (n)	Adjuvant reirradiation (n)	Definitive reirradiation (n)	ORN cases (n)	ORN Reporting System
Al-Wassia et al. 2015	27	12	14	5	CTCAEv3
Awan et al. 2018	46	32	13	0	CTCAEv3
Bhalavat et al. 2018	14	0	14	0	RTOG
Bahl et al. 2018	20	0	20	0	CTCAEv3
Beddok et al. 2023	23	9	14	5	CTCAEv5
Benchalal et al. 1995	19	14	5	1	self-defined
Berger et al. 2010	57	0	57	0	CTCAEv3
Bots et al. 2017	137	108	29	5	RTOG
Boustani et al. 2021	33	0	33	0	CTCAEv5
Buglione et al. 2015	75	11	64	1	CTCAEv4
Bussu et al. 2024	34	29	5	0	CTCAEv5
Cengiz et al. 2011	46	0	46	1	RTOG
Chen et al. 2007	113	113	0	0	NR
Chen et al. 2011	21	0	21	0	RTOG
Crevoisier et al. 2001	25	25	0	4	RTOG
Cvek et al. 2016	40	0	40	4	RTOG
Dionisi et al. 2019	17	0	16	0	CTCAEv5
Dornoff et al. 2015	66	16	50	0	CTCAEv3/v4
Duprez et al. 2009	84	19	65	2	LENT-SOMA
Embring et al. 2021	54	22	32	3	LENT-SOMA
Hall et al. 2003	18	18	0	0	RTOG
Hegde et al. 2018	20	5	15	1	CTCAEv4
Hepel et al. 2005	30	11	19	1	NR
Held et al. 2019	229	39	190	3	CTCAEv4
Heron et al. 2009	25	0	25	0	NR
Hsieh et al. 2024	22	22	0	4	CTCAEv5
Hoell et al. 2023	45			1	CTCAE
Hwang et al. 1998	64	8	56	1	RTOG
Jiang et al. 2019	61	0	61	0	RTOG
Jensen et al. 2010	21	0	21	0	CTCv3
Kakria et al. 2014	31	4	27	0	CTCAEv3
Karam et al. 2016	42	12	30	0	RTOG
Kramer et al. 2005	38	0	38	2	CTCv2
Lee et al. 2017	20	0	20	0	CTCAEv4
Lee et al. 2016	66	25	41	2	CTCAEv4
Low et al. 2006	36	0	36	0	NR
Megahed et al. 2024	9	8	1	0	CTCAEv5
Martínez-Fernández et al. 2017	63	63	0	3	RTOG
Orlandi et al. 2019	159	60	99	2	CTCAE
Ozyigit et al. 2011	27	0	27	0	CTCAEv3
Pialat et al. 2021	25	22	3	0	CTCv2
Ritter et al. 2016	15	15	0	0	LENT-SOMA
Roh et al. 2009	36	0	36	1	RTOG
Romesser et al. 2016	92	36	56	1	CTCAEv4/RTOG
Rühle et al. 2020	48	17	31	2	CTCAEv4
Rudzianskas et al. 2014	63	31	32	3	RTOG
Schaefer et al. 2000	32	0	32	0	RTOG
Salama et al. 2006	115	49	66	13	CTCAEv3
Seiwert et al. 2008	29			3	CTCv2
Siddiqui et al. 2009	21	0	21	1	RTOG
Spencer et al. 2007	79	0	79	0	RTOG
Suh et al. 2008	12	12	0	1	RTOG
Sulman et al. 2009	74	20	54	3	self-defined
Takiar et al. 2016	206	104	102	5	CTCAEv4
Teo et al. 1998	117	14	103	1	self-defined
Tselis et al. 2011	74	5	69	1	RTOG
Uysal et al. 2023	10	0	10	0	CTCAEv5
Vischioni et al. 2020	51	0	51	1	CTCAEv4
Voynov et al. 2006	22	0	22	0	CTCv2
Waldron et al. 2019	26	26	0	1	CTCAEv4
Zheng et al. 2004	117	0	117	1	RTOG
Zwicker et al. 2011	38	13	25	0	CTCAEv3

Abbreviations: CTC, Common Toxicity Criteria; CTCAE, Common Terminology Criteria for Adverse Events; LENT-SOMA, Late Effects Normal Tissue – Subjective, Objective, Management, Analytic; NR, not reported; ORN, osteoradionecrosis; RTOG, Radiation Therapy Oncology Group.

In terms of radiation modalities, teletherapy was applied in 2546 patients and brachytherapy in 684 patients. Photon-based radiotherapy was used in 2730 patients, while proton therapy (*n* = 141), heavy ion therapy (*n* = 284), and electron therapy (*n* = 117) were less frequently applied. Due to the use of combined or sequential radiation modalities in some studies, the total number of reported treatment modalities exceeds the total number of included patients. The most commonly used radiation technique was IMRT (*n* = 1042).

The pooled median interval between initial radiotherapy and reirradiation was 29.5 months (range, 0–756), the pooled median reirradiation dose was 60 Gy (range, 4–108), and the pooled median initial radiotherapy dose was 65 Gy (range, 15–134).

### 3.4 Incidence of ORN

The pooled median follow-up duration was 26 months (range, 1–180 months) among surviving patients and 20 months (range, 0–308 months) for the overall cohort. The overall observed incidence of ORN of the mandible and/or maxilla was 3.6% (95% CI, 2.8%–4.6%) across the included studies, with moderate statistical heterogeneity (I^2^ = 37.5%; τ^2^ = 0.495) (Fig. 2). The corresponding 95% prediction interval (PI) was 0.9%–13.4%. When restricted to patients with available follow-up for late adverse events, the observed incidence of ORN was 4.0% (95% CI, 3.0%–5.3%, I^2^ = 36.0%; τ^2^ = 0.472, 95% PI, 1.0%–14.5%). The pooled observed incidence of ORN across all anatomical sites, including non-jaw osseous sites, was 4.7% (95% CI, 3.6%–6.1%, I^2^ = 42.4%; τ^2^ = 0.477, 95% PI, 1.2%–16.8%) in the total population of patients receiving reirradiation (Supplementary Material I). When restricted to studies explicitly reporting mandibular ORN (as opposed to ORN of the jaw), the pooled observed incidence was 3.5% (95% CI, 2.7%–4.6%, I^2^ = 37.4%; τ^2^ = 0.536, 95% PI, 0.8%–14.0%) (Supplementary Material I). Fourteen out of the 62 included studies reported ORN at non-mandibular sites such as cervical vertebra, skull base, and hyoid bone.

**Fig. 2:**
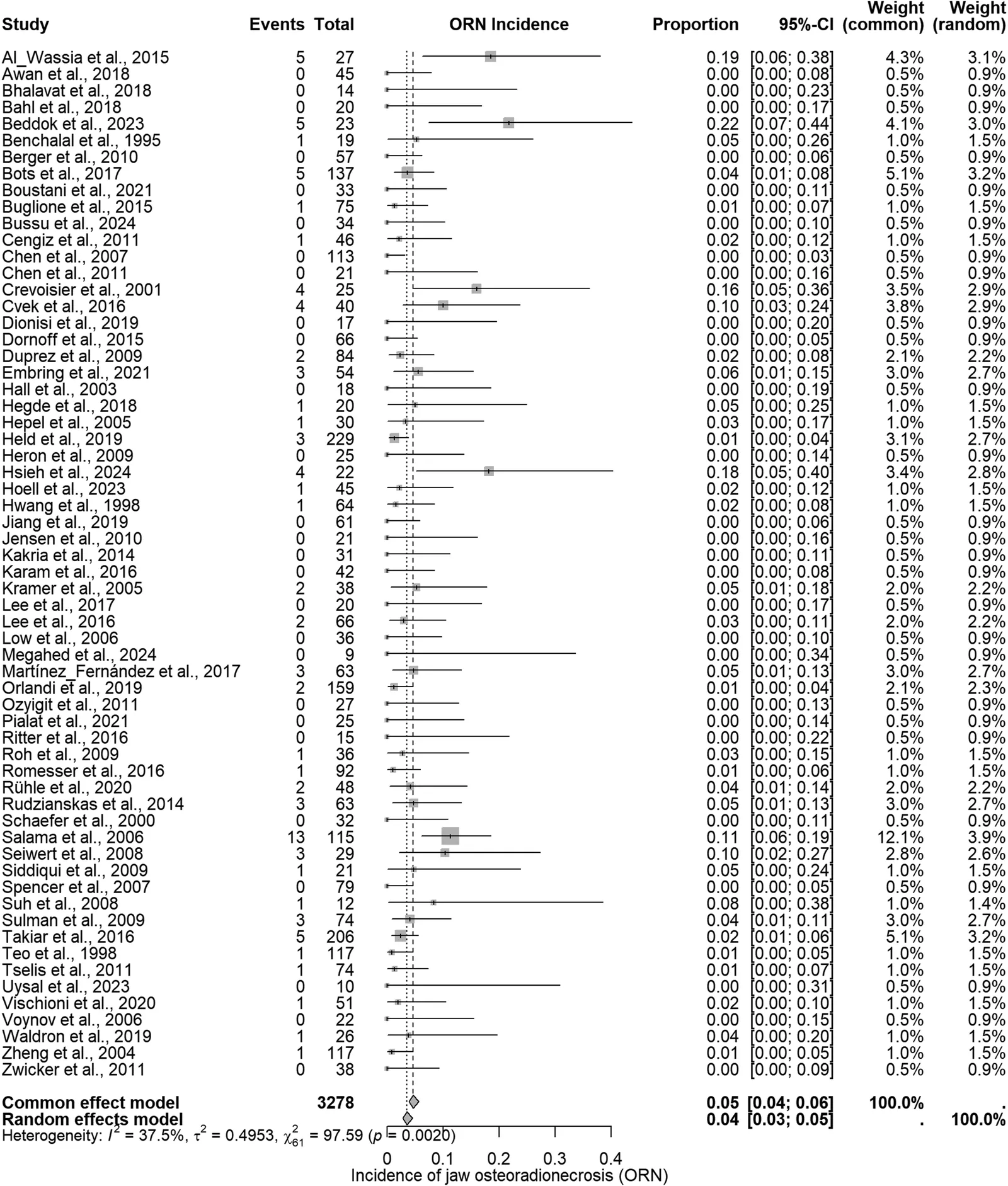
Pooled observed incidence of osteoradionecrosis (ORN) of the mandible or maxilla after reirradiation of recurrent or second primary head and neck cancer.

### 3.5 Severity of ORN

Of the 89 ORN cases reported across the included studies, 42 cases from 18 studies were reported with individual grades, 40 cases from 13 studies were reported in cumulative grade categories only, and 7 cases from 4 studies were reported without grading information (Table 3). Among individually graded cases, grade 4 ORN represented the most frequently reported severity level (20 of 42 cases, 47.6%), and grade ≥ 3 ORN accounted for 35 of 42 cases (83.3%). ORN as a late adverse event was most commonly reported using CTCAE, which was applied in 33 studies. Other classification systems included the RTOG criteria (19 studies), the LENT-SOMA scale (3 studies), and study-specific classifications (3 studies), while 4 studies did not report the classification system used.

**Table 3:** Severity of osteoradionecrosis (ORN) according to the availability of grading information.

Studies with detailed grading information (*n* = 18)	ORN cases (*n* = 42)
Grade 1	3 (7.1%)
Grade 2	4 (9.5%)
Grade 3	15 (35.7%)
Grade 4	20 (47.6%)
Grade 5	0 (0.0%)
**Studies with cumulative grading information (*****n*** **= 13)**	**ORN cases (*n* = 40)**
Grade 1–2	5 (12.5%)
Grade 3–4	11 (27.5%)
Grade 3–5	11 (27.5%)
Grade 4–5	13 (32.5%)
**Studies with no grading information (n = 4)**	**ORN cases (*n* = 7)**

Percentages refer to the number of ORN cases within each category. Abbreviations: ORN, osteoradionecrosis.

### 3.6 Determinants of ORN

In the univariable meta-regression analysis, no significant association was observed between the pooled median reirradiation dose and the observed incidence of ORN (β = 0.013; *P* = .355). In the multivariable meta-regression including both initial and reirradiation dose, neither the dose of the initial radiotherapy (β = −0.018; *P* = .677) nor the reirradiation dose (β = 0.033; *P* = .077) showed a statistically significant association with observed ORN incidence.

The meta-regression analysis including radiation modality as a moderator (reference: photon therapy) demonstrated no significant association for proton therapy (β = 0.557; *P* = .400), heavy ion therapy (β = −0.923; *P* = .207), or electron therapy (β = −2.169; *P* = .169) with the observed ORN incidence. Similarly, the pooled median interval between initial radiotherapy and reirradiation was not significantly associated with observed ORN incidence (β = −0.005; 95% CI, −0.024 to 0.014; *P* = .620). In contrast, a higher proportion of patients undergoing adjuvant reirradiation versus definitive reirradiation was associated with an increased observed incidence of ORN (β = 1.091; 95% CI, 0.256–1.926; *P* = .01).

### 3.7 Assessment of publication bias

Visual inspection of the funnel plot suggested asymmetry, and Egger's regression test indicated significant funnel plot asymmetry (bias coefficient − 1.815, SE = 0.289; *t* = −6.28, df = 60, *P* < .0001) (Fig. 3). The regression model accounted for between-study heterogeneity using a multiplicative residual variance approach (residual variance parameter = 0.9813).

**Fig. 3:**
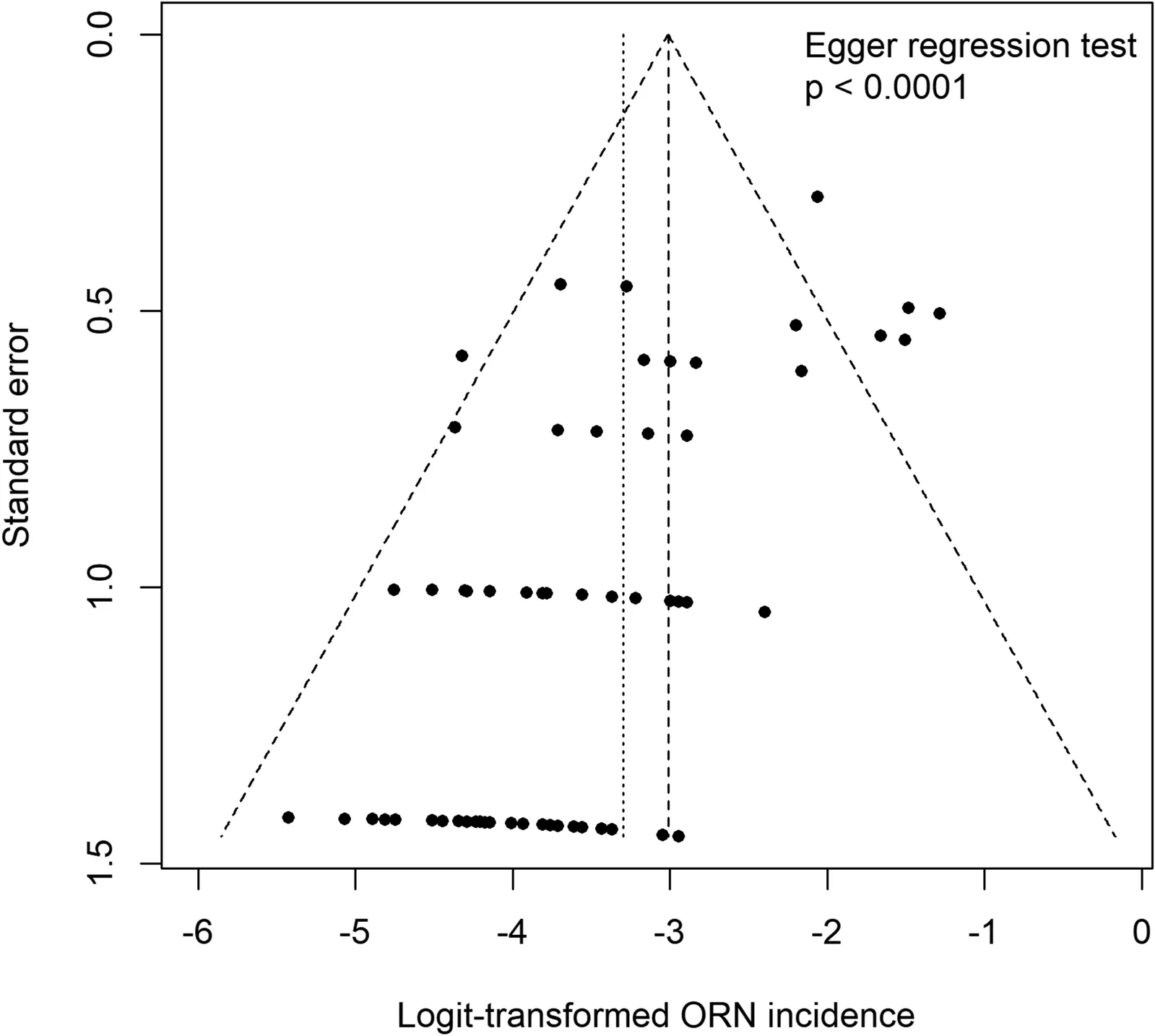
Funnel plot of osteoradionecrosis (ORN) observed incidence, reported for the maxilla and mandible.

## 4 Discussion

To our knowledge, this is the first systematic review and meta-analysis specifically evaluating the observed incidence of ORN following reirradiation in the head and neck region. In this analysis, the pooled observed incidence of ORN ranged from 3.5% to 4.7%, depending on the definition and population considered. Among ORN cases with available grading information, grade 4 represented the most common severity level. These findings provide a clinically relevant benchmark for estimating ORN risk in this setting and may inform patient counseling.

The observed incidence of ORN in the reirradiation setting, as well as the corresponding prediction intervals, fall within the range reported after a single course of radiotherapy, where rates typically range between approximately 2% and 15% depending on patient population, treatment technique, and follow-up duration [10], [24], [25], [26]. The comparable incidence observed in this analysis, despite cumulative radiation exposure, may be explained by several factors. First, competing events such as mortality in this high-risk population likely lead to an underestimation of late adverse events such as ORN in long-term survivors [13]. This is especially relevant concerning the substantial variation in follow-up duration across included studies. Second, reirradiation is often delivered to smaller target volumes without elective nodal irradiation, potentially reducing the dose to osseous structures. Especially in patients with recurrent or second primary HNC outside the oral cavity and oropharynx, the cumulative radiation dose to the mandible can be reduced when only the recurrent tumor with a small margin is irradiated. Third, patient selection may have contributed, as individuals with pre-existing ORN were excluded, although subclinical disease cannot be entirely ruled out.

The meta-regression demonstrated a trend toward a higher observed incidence of ORN with increasing reirradiation dose (β = 0.033; *P* = .077), although this association did not reach statistical significance. Similarly, neither the dose of the initial radiotherapy nor the interval between radiation courses was significantly associated with observed ORN incidence. These findings suggest that, in addition to radiation dose, factors such as patient characteristics, tumor location, surgical interventions, and individual tissue susceptibility likely contribute to ORN risk. Nevertheless, based on radiobiological and pathophysiological considerations, as well as evidence from the primary radiotherapy setting, a dose–response relationship between mandibular radiation dose and the risk of ORN after reirradiation remains biologically plausible. Reported cumulative dose thresholds for ORN in the reirradiation setting range from approximately 114 Gy to 119 Gy, although available data remain limited and heterogeneous [13], [27]. These thresholds have also been incorporated into the recent international Reirradiation Collaborative Group (ReCOG)/ESTRO/ASTRO expert consensus recommendations, which advise keeping the cumulative EQD2 to the mandible below approximately 114–119 Gy whenever feasible [28]. An analysis of different treatment modalities, including proton, heavy ion, and electron therapy, showed no association with observed ORN incidence, but was limited by smaller case numbers and was therefore insufficient to determine a definitive conclusion.

In contrast, the respective setting of reirradiation was identified as a significant study-level determinant, with a higher proportion of adjuvant reirradiation being associated with a higher observed incidence of ORN compared to definitive treatment. This may reflect differences in patient selection, surgical history, dose distribution, tumor location, or cumulative treatment burden, although this finding should be interpreted with caution. This observation may be explained by longer survival in patients undergoing adjuvant reirradiation, allowing more time for late adverse events to manifest, as suggested by previous analyses [29]. In addition, adjuvant treatment is also often delivered to larger target volumes than definitive reirradiation; however, dose distribution differences to the mandible or maxilla between settings remain unclear and warrant future dosimetric study. Overall, this association should be regarded as hypothesis-generating rather than causal.

The overall observed incidence of ORN in this study, although clinically relevant, does not appear disproportionately high when compared to other severe late adverse events associated with reirradiation, such as high-grade dysphagia, mucosal necrosis, vascular complications including carotid blowout syndrome, or neurologic injury (e.g., plexopathy, brainstem necrosis, or temporal lobe necrosis) [30], [31], [32]. The observed incidence estimates may provide a useful reference for future evaluations of mandibular dose constraints in the reirradiation setting; however, the present data do not permit conclusions regarding the adequacy of existing dose constraints. Possible preventive measures primarily include pre-radiotherapy dental optimization, maintenance of good oral hygiene, avoidance of post-treatment dental extractions whenever possible, and regular dental surveillance [12]. Future studies are needed to specifically evaluate whether current preventive and surveillance strategies in the reirradiation setting might require intensification.

The assessment of ORN severity revealed that high-grade adverse events, particularly grade 4 ORN, accounted for a substantial proportion of reported cases, highlighting the clinical relevance of ORN as a severe and potentially debilitating late complication. Although this distribution may have been influenced by underdetection and underreporting of early-stage ORN, particularly in the absence of systematic screening or standardized diagnostic criteria, the predominance of severe cases underscores the importance of vigilant surveillance and timely diagnosis in patients undergoing reirradiation.

In addition, information on the management of ORN was largely lacking in the included studies. Details regarding treatment strategies, including surgical interventions, pharmacological approaches such as pentoxifylline and tocopherol, or the use of adjunctive therapies such as hyperbaric oxygen, were rarely reported. This limits the ability to draw conclusions on optimal management and may further reflect the inconsistent documentation of ORN as a secondary outcome in the reirradiation setting.

The funnel plot asymmetry and the significant Egger's test should not be interpreted as evidence of publication bias alone. In proportion meta-analyses, asymmetry may also result from true between-study heterogeneity (e.g., in patient populations, techniques, ORN definitions and follow-up), from floor effects inherent to a low-incidence outcome, from zero- or few-event studies requiring continuity corrections, and from genuine small-study effects such as more thorough adverse event documentation in smaller single-institution series. The observed asymmetry therefore most likely reflects a combination of these factors, although selective reporting of ORN as a secondary outcome cannot be excluded.

### 4.1 Limitations

Several limitations should be considered when interpreting these results. First, ORN was frequently reported as a secondary outcome, and in many studies, detailed information on occurrence, localization, and grading was incomplete. Second, substantial heterogeneity existed in the definition and classification of ORN across studies, which likely contributed to between-study heterogeneity, and limits direct comparability of pooled estimates. Third, mixed patient populations and inconsistent reporting of reirradiated subgroups required additional data retrieval and may have introduced bias. Fourth, the diagnosis of ORN is inherently challenging and may be subject to underreporting. ORN may be overlooked in the absence of systematic screening or standardized diagnostic criteria, particularly in retrospective analyses. This is especially relevant in early-stage disease, which may remain asymptomatic and therefore undetected in the absence of clinical signs such as pain, exposed bone, or swelling, or if patients do not undergo regular dental follow-up. Further, a lack of sufficiently detailed time-to-event or individual patient data did not allow estimation of a formal cumulative incidence, e.g., including adjustments for competing risk or incomplete follow-up, rather than our estimated observed incidence. Likewise, time to ORN onset and follow-up duration among patients developing ORN were rarely reported, as ORN was mostly documented among other adverse events rather than as a primary outcome. Taken together, these findings underscore the need for standardized reporting of late adverse events, including ORN, in future studies on reirradiation [28]. The wide time span of included studies (1995–2024) introduces potential heterogeneity related to technical advances (e.g., the transition from conventional/3D-conformal radiotherapy to IMRT, proton, and heavy-ion therapy) and patient selection practices over time. A temporal reduction in ORN risk with modern techniques, while biologically plausible, cannot be confirmed or excluded by the present data. Further, studies published after the search cut-off date (March 31, 2025) were not systematically captured. Therefore, our findings should be interpreted considering the evolving landscape of reirradiation techniques.

## 5 Conclusion

In this systematic review and meta-analysis, the observed incidence of ORN following reirradiation was clinically relevant, with a considerable proportion of severe cases. A higher study-level proportion of adjuvant reirradiation was associated with higher observed ORN incidence. Future studies should aim for standardized assessment and reporting of ORN, with particular emphasis on the systematic detection and documentation of early-stage disease, to improve comparability and enable more precise risk estimation.

## Data sharing

Research data are stored in an institutional repository and will be shared upon request to the corresponding author.

## CRediT authorship contribution statement

**Annemarie Fritz:** Writing – review & editing, Writing – original draft, Validation, Software, Methodology, Investigation, Formal analysis, Data curation, Conceptualization. **Nils Gleim:** Writing – review & editing, Software, Investigation, Formal analysis, Data curation. **Anita Kloss-Brandstätter:** Writing – review & editing, Visualization, Investigation. **Thomas Kuhnt:** Writing – review & editing, Conceptualization. **Nils H. Nicolay:** Writing – review & editing, Conceptualization. **Andreas Maximilian Fichter:** Writing – review & editing, Supervision, Project administration. **Sebastian Gaus:** Writing – review & editing, Supervision, Methodology, Investigation, Data curation, Conceptualization. **Alexander Rühle:** Writing – review & editing, Writing – original draft, Validation, Supervision, Resources, Project administration, Methodology, Investigation, Funding acquisition, Data curation, Conceptualization.

## Ethics approval

The Ethics Committee of the University of Leipzig provided a waiver for this meta-analysis on February 20, 2025.

## Ethics declaration

The authors have NOT obtained informed consent from participants or their legal representatives. This systematic review and meta-analysis used only aggregated data from previously published studies; no individual patient data were collected and no patients were contacted, so informed consent was not required.

This study was performed in compliance with relevant laws, regulatory frameworks and guidelines where the research took place. The Ethics Committee of the University of Leipzig granted an exemption. The authors provided the following explanation: The Ethics Committee of the University of Leipzig provided a waiver for this study on February 20, 2025.

## Funding

This project received partial funding from the *Roland-Ernst-Stiftung für Gesundheitswesen*. The funding sources had no role in the design and conduct of the study; collection, management, analysis, and interpretation of the data; preparation, review, or approval of the manuscript; or decision to submit the manuscript for publication.

## Declaration of competing interest

The authors declare the following financial interests/personal relationships which may be considered as potential competing interests: The authors declare that they have no known competing financial interests or personal relationships that could have appeared to influence the work reported in this paper.
