# Supplementary material for: Osteoradionecrosis in patients with head and neck cancer after reirradiation – a systematic review and meta-analysis
Source: Clin Transl Radiat Oncol. 2026 Oct 28;62:101342. doi: 10.1016/j.ctro.2026.101342 (PMC13744302; doi:10.1016/j.ctro.2026.101342)
Supplement: Supplementary file 1 [file mmc2.pdf]

Supplementary Material

Osteoradionecrosis in Patients with Head and Neck Cancer After Reirradiation: A Systematic Review and Meta-Analysis

Table of Contents

Supplementary (A): PROSPERO protocol p. 2

Supplementary (B): Search strategies p. 9

Supplementary (C): Inclusion and exclusion criteria p. 10

Supplementary (E): Late toxicity estimation p. 11

Supplementary (F): Risk of bias assessment p. 12

Supplementary (G): Excludes studies with reason p. 16

Supplementary (H): Risk of bias assessment – full assessment table p. 30

Supplementary (I): Overall incidence of ORN p. 34

Supplementary (A): PROSPERO protocol

NIHR | National Institute for Health and Care Research

PROSPERO
International prospective register of systematic reviews

Cumulative Incidence of Osteoradionecrosis in Patients with Recurrent or Secondary Primary Head and Neck Cancer Undergoing Reirradiation: A Systematic Review with Meta-analysis

Annemarie Fritz, Sebastian Gaus, Anita Kloss-Brandstätter, Thomas Kuhnt, Nils H Nicolay, Andreas Fichter, Alexander Rühle

To enable PROSPERO to focus on COVID-19 submissions, this registration record has undergone basic automated checks for eligibility and is published exactly as submitted. PROSPERO has never provided peer review, and usual checking by the PROSPERO team does not endorse content. Therefore, automatically published records should be treated as any other PROSPERO registration. Further detail is provided here.

Citation 1 change
Annemarie Fritz, Sebastian Gaus, Anita Kloss-Brandstätter, Thomas Kuhnt, Nils H Nicolay, Andreas Fichter, Alexander Rühle. Cumulative Incidence of Osteoradionecrosis in Patients with Recurrent or Secondary Primary Head and Neck Cancer Undergoing Reirradiation: A Systematic Review with Meta-analysis. PROSPERO 2025 CRD42025635226. Available from https://www.crd.york.ac.uk/PROSPERO/view/CRD42025635226.

REVIEW TITLE AND BASIC DETAILS

Review title

Cumulative Incidence of Osteoradionecrosis in Patients with Recurrent or Secondary Primary Head and Neck Cancer Undergoing Reirradiation: A Systematic Review with Meta-analysis

Review objectives

What is the incidence of osteoradionecrosis in patients with recurrent or second primary head-and-neck cancer after reirradiation?

Keywords

brachytherapy, head-and-neck cancer, head-and-neck squamous cell carcinoma, ORN,

Osteoradionecrosis, radiotherapy, reirradiation, re-irradiation

SEARCHING AND SCREENING

Searches 1 change
PubMed/MEDLINEWeb of ScienceReference lists/bibliographies of included studiesArticle language: English, German

Publication date: Articles published since January 01, 1995

Study design 1 change
Both randomized and nonrandomized study types will be included.

Included
Randomized clinical trial and observational studies (cohort, case-control) with any follow up period after reirradiation and full text available (English or German language)

Excluded
Case series without consecutive inclusion mentioned, study protocol, conference abstracts, other language than English or German, toxicities not reported

Link to search strategy 1 change
A full search strategy is available in the full protocol as described in the *Availability of full protocol* section

ELIGIBILITY CRITERIA

Condition or domain being studied
Osteoradionecrosis remains one of the most challenging complications associated with radiotherapy of head-and-neck cancer. This late-onset condition profoundly affects the quality of life of cancer patients and survivors, often persisting long after treatment. Clinically, osteoradionecrosis is characterized by exposed or necrotic bone that may become superinfected and is often accompanied by symptoms such as oral or extraoral fistulas, pain, trismus, facial numbness, and deformities. The underlying pathophysiology of osteoradionecrosis is primarily attributed to radiotherapy-induced vascular damage, which leads to reduced blood supply and subsequent bone necrosis. The mandible is particularly susceptible due to its relatively poor vascularity.
The incidence of osteoradionecrosis is higher in patients undergoing reirradiation compared with those receiving only one course of radiotherapy. However, the precise rates of osteoradionecrosis in patients undergoing reirradiation are unknown.

Population 1 change

Included
Adult patients (18 years and older) with locally/locoregionally recurrent or secondary primary cancer of the head and neck region

Intervention(s) or exposure(s)

Reirradiation (tele- and brachytherapy allowed) for head and neck cancer with or without neoadjuvant, current, or adjuvant systemic anti-cancer treatment with or without surgery
Excluded:
Patients with the first course of radiotherapy within the head and neck area
Patients treated with chemotherapy or surgery without radiotherapy

Comparator(s) or control(s)

No control group

OUTCOMES TO BE ANALYSED

Main outcomes

Diagnosis of osteoradionecrosis and indication of frequency, measured in one of the following ways:
1. Reported outcome in the study
2. If not reported: Diagnosis provided upon written request to the corresponding author

Measures of effect
If the primary outcome of interest is reported in more than 10 independent studies, a meta-analysis will be performed. Quantitative data from included studies will be synthesized using meta-analytic techniques, where appropriate. The primary analysis will calculate pooled effect estimates (e.g., risk ratios, odds ratios, mean differences, or standardized mean differences) using random-effects models to account for heterogeneity between studies, with results displayed in forest plots.

Additional outcomes

Not applicable

Measures of effect

DATA COLLECTION PROCESS

Data extraction (selection and coding)

Step 1: Two reviewers will independently review the identified electronic database citations for the listed inclusion/exclusion criteria. The studies found are collected in the reference management software Citavi.
Step 2: Third reviewer will review the above independent screening assessments and finalize the studies to be included based on the listed inclusion/exclusion criteria.
Step 3: Creating a four-phase flow diagram according to PRISMA to provide an overview of included and excluded studies.
Step 4: Critical appraisal of all included studies by two reviewers independently
Step 5: For all of the included studies, the following information will be recorded in excel

spreadsheets:
Year of publication
Title
First author
Country of analyzed treatment center(s)
Funding Source/ Conflict of Interest
Type of study
Sample size
Inclusion period
Gender
Sex
Patient age
Smoking history
Cancer histology
p16/HPV status
Tumor localization at time of reirradiation
Recurrent vs. second primary carcinoma
TNM stage
Grading
Treatment protocol (Re-RT adjuvante, definitve, palliative)
Radiation technique
Type of radiation (photons, protons, electrons, heavy ions)
Time interval between initial course of radiotherapy and reirradiation
Number of fractions of the initial course of radiotherapy
Radiation dose of the initial course of radiotherapy
Number of fractions of the reirradiation course
Radiation dose of the reirradiation course
Cumulative radiation dose
Cumulative radiation dose to the mandible
Time of follow-up after reirradiation
Frequency of osteoradionecrosis
Osteoradionecrosis classification/grading
Time until development of osteoradionecrosis
The reviewers will contact the authors of the studies to clarify existing data, request missing information, or obtain additional data as needed.
Step 6: Synthesize evidence into tables

Risk of bias (quality) assessment

Two reviewers will independently assess included articles for Risk of Bias according to their study design:
Randomized controlled Trials: Cochrane Risk of Bias Tool (RoB 2)
Non-randomized Studies of Interventions: ROBINS-I
Non-randomized controlled trials: Newcastle-Ottawa Scale (NOS)

Cross-sectional studies: JBI Critical Appraisal Checklist for Analytical Cross Sectional Studies
In order to assess meta-biases, we plan to compare protocols of any registered study with published reports to detect selective non-reporting of results (outcome reporting bias). In addition, we plan to use the random effects model to assess the possible presence of small sample bias and to take possible heterogeneity into consideration. To evaluate the risk of publication bias, a funnel plot will be visually inspected for asymmetry, and statistical tests (Egger's test and Begg's test) will be applied when at least 10 studies are included. Sensitivity analyses will be performed to assess the robustness of the findings by excluding studies with high risk of bias or using alternative statistical models.

PLANNED DATA SYNTHESIS

Strategy for data synthesis

To evaluate possible risk factors for osteoradionecrosis, additional data will be extracted from the included studies where these factors are reported. For each risk factor, pooled effect estimates (e.g., odds ratios or risk ratios) will be calculated using random-effects models. If sufficient data are available, meta-regression will be used to investigate the association of these risk factors with osteoradionecrosis rates. Subgroup analyses will also be conducted to explore the impact of key risk factors, such as radiation dose, tumor location, and concurrent treatments. These analyses will provide insight into the relative contribution of specific risk factors to the development of osteoradionecrosis in the context of reirradiation.
Study heterogeneity will be assessed using the I^2 statistic, I^2 -values of 25%, 50%, and 75% will be interpreted as indicating low, moderate, and high heterogeneity, respectively. Where substantial heterogeneity (I^2 >50) is detected, subgroup analyses and meta-regression will be conducted to explore potential sources of heterogeneity.

Analysis of subgroups or subsets

Where data permit, subgroup analyses (e.g. patients undergoing brachytherapy alone; patients receiving radical reirradiation without previous surgical resection) will be conducted. Meta-regression will be used to explore the impact of continuous moderators on effect sizes.

REVIEW AFFILIATION, FUNDING AND PEER REVIEW

Review team members

- Dr Annemarie Fritz, University Medical Center Leipzig
- Dr Sebastian Gaus, University Medical Center Leipzig
- Dr Anita Kloss-Brandstätter, Carinthia University of Applied Sciences
- Professor Thomas Kuhnt, University Medical Center Leipzig
- Professor Nils H Nicolay, University Medical Center Leipzig
- Professor Andreas Fichter, University Medical Center Leipzig
- Dr Alexander Rühle, University Medical Center Leipzig

Review affiliation

University Medical Center Leipzig

Funding source

Roland-Ernst-Stiftung für Gesundheitswesen

Named contact

Alexander Rühle. University Medical Center Leipzig\\Stephanstr. 9a\\04103 Leipzig
alexander.ruehle@medizin.uni-leipzig.de

TIMELINE OF THE REVIEW

Review timeline

Start date: 01 February 2025. End date: 30 June 2025

Date of first submission to PROSPERO 1 change

20 January 2025

Date of registration in PROSPERO 1 change

31 January 2025

AVAILABILITY OF FULL PROTOCOL

Availability of full protocol 1 change

A full protocol has been written and uploaded to PROSPERO. The protocol may be accessed through this link https://www.crd.york.ac.uk/PROSPEROFILES/2284f3ece4dac3cb52b3f90b05b2da6a.pdf.

CURRENT REVIEW STAGE

Publication of review results

The intention is to publish the review once completed.The review will be published in English

Stage of the review at this submission 1 change

Review stage	Started	Completed
Pilot work	✓	✓
Formal searching/study identification	✓	✓
Screening search results against inclusion criteria	✓	✓
Data extraction or receipt of IPD	✓	✓
Risk of bias/quality assessment	✓	✓
Data synthesis	✓	✓

Review status

The review is currently planned or ongoing.

ADDITIONAL INFORMATION

PROSPERO version history 1 change

- Version 2.0, published 01 Jun 2025
- Version 1.1, published 31 Jan 2025
- Version 1.0, published 31 Jan 2025

Review conflict of interest

None known

Country

Germany

Revision note 1 change

- Inclusion criterion added: Only studies published since January 01, 1995 will be included

- Exclusion criterion changed: Case series without consecutive inclusion mentioned

- Population: "locally/locoregionally" added

Disclaimer 1 change

The content of this record displays the information provided by the review team. PROSPERO does not peer review registration records or endorse their content.

PROSPERO accepts and posts the information provided in good faith; responsibility for record content rests with the review team. The guarantor for this record has affirmed that the information provided is truthful and that they understand that deliberate provision of inaccurate information may be construed as scientific misconduct.

PROSPERO does not accept any liability for the content provided in this record or for its use. Readers use the information provided in this record at their own risk.

Any enquiries about the record should be referred to the named review contact

Supplementary Material (B): Search strategies

PubMed

("head and neck cancer"[MeSH Terms] OR "head and neck neoplasms"[MeSH Terms] OR "head and neck cancer" OR "head and neck tumor" OR "oropharyngeal cancer" OR "laryngeal cancer" OR "nasopharyngeal cancer" OR "oral cancer")
AND
("recurrent" OR "secondary" OR "relapse" OR "locally recurrent" OR "metachronous")
AND
("reirradiation" OR "re-irradiation" OR "repeat radiotherapy" OR "salvage radiotherapy")
AND
("osteoradionecrosis" OR "ORN" OR "toxicity" OR "side effects" OR "complications")

Filters used:
Article language: English, German
Publication date custom range: From 1995/1/1

Web of Sience

TS = ("head and neck cancer" OR "head and neck tumor" OR "oropharyngeal cancer" OR "laryngeal cancer" OR "nasopharyngeal cancer" OR "oral cancer")
AND TS = ("recurrent" OR "secondary" OR "locally recurrent" OR "metachronous" OR "relapse")
AND TS = ("re-irradiation" OR "reirradiation" OR "salvage radiotherapy" OR "repeat radiotherapy")
AND TS = ("toxicity" OR "side effects" OR "adverse effects" OR "treatment-related toxicity" OR "complications" OR "adverse events" OR "radiation-induced toxicity" OR "late effects" OR "treatment toxicity" OR "osteoradionecrosis" OR "bone necrosis" OR "mandibular necrosis" OR "radiation necrosis")

Filters used:
Languages: English, German
Publication date: From 1995/1/1

Supplementary Material (C): Inclusion and exclusion criteria

Inclusion criteria

- Occurrence of ORN as an Adverse Event (AE) after reirradiation; Common Terminology Criteria for Adverse Events (CTCAE) Grade 1 up to Grade 5
- Reirradiation for head and neck cancer with or without neoadjuvant, current, or adjuvant systemic anti-cancer treatment with or without surgery; Tele- and brachytherapy
- Adult patients (≥18 years) with recurrent or secondary primary cancer of the head and neck region
- Randomized clinical trial and observational studies (cohort, case-control) with any follow up period after reirradiation and full text available
- Publication date: Articles published since January 01, 1995
- English or German language

Exclusion criteria

- ORN that was present prior to reirradiation
- ORN not involving maxilla and/or mandible
- Patients with first course of radiotherapy within the head and neck area
- Patients treated with chemotherapy or surgery without radiotherapy
- Paediatric
- Cancers not located in the head and neck region
- Case series without consecutive inclusion mentioned
- Study protocols and conference abstracts
- No information provided regarding ORN
- No toxicity reported
- Other language than English or German

Supplementary Material (E): Late toxicity estimation

In cases where the number of patients available for the evaluation of osteoradionecrosis (ORN) as a late toxicity (defined as occurring ≥ 90 days after reirradiation) was not explicitly reported, the predefined estimation method *IPDfromKM* according to Liu et al. was used [1]. In this approach, Kaplan-Meier (KM) curves are visually analyzed to extract individual patient data (IPD), using the package *IPDfromKM* in R statistical software [2].

Of the 62 studies included, the number of patients available for late toxicity evaluation was estimated using *IPDfromKM* for 30 studies. In total, this patient number deviated from the initial patient number in 40/62 studies, including those providing detailed information on late toxicity. Incidences of ORN were estimated separately for all patients and only for those with follow-up available for late toxicity evaluation, respectively.

[1] Liu N, Zhou Y, Lee JJ. IPDfromKM: reconstruct individual patient data from published Kaplan-Meier survival curves. BMC Med Res Methodol. 2021 Jun 1;21(1):111. doi: 10.1186/s12874-021-01308-8. PMID: 34074267; PMCID: PMC8168323.

[2] R Core Team (2024). R: A Language and Environment for Statistical Computing. R Foundation for Statistical Computing, Vienna, Austria. https://www.R-project.org/.

Supplementary Material (F): Risk of bias assessment

Methodological items for non-randomized studies (MINORS) instrument

Items are scored as 0 (not reported), 1 (reported but inadequate), or 2 (reported and adequate). The maximum score for non-comparative studies is 16, and for comparative studies is 24.
For improved comparability and integration into the statistical analysis, scores were converted to a percentage scale by multiplying by 100.

(1) A clearly stated aim: The question addressed should be precise and relevant in the light of available literature.
(2) Inclusion of consecutive patients: All patients potentially fir for inclusion (satisfying the criteria for inclusion) have been included in the study during the study period.
(3) Prospective collection of data: Data were collected according to a protocol established before the beginning of the study.
(4) Endpoints appropriate to the aim of the study: Unambiguous explanation of the criteria used to evaluate the main outcome, which should be in accordance with the question addressed by the study. Also, the endpoints should be assessed on an intention-to-treat basis.
(5) Unbiased assessment of the study endpoint: Blind evaluation of objective endpoints and double-blind evaluation of subjective endpoints. Otherwise the reasons for not blinding should be stated.
(6) (6) Follow-up period appropriate to the aim of the study: The follow-up should be sufficiently long to allow the assessment of the main endpoint and possible adverse events.
(7) Loss to follow up less than 5%: All patient should be included in the follow up. Otherwise, the proportion lost to follow up should not exceed the proportion experiencing the major endpoint.
(8) Prospective calculation of study size: Information of the size of detectable difference of interest with a calculation of 95% confidence interval, according to the expected incidence of the outcome event, and information about the level for statistical significance and estimates of power when comparing outcomes.

Additional criteria in the case of comparative studies:
(9) An adequate control group: Having a gold standard diagnostic test or therapeutic intervention recognized as the optimal intervention according to the available published data.
(10) Contemporary groups: Control and studied group should be managed during the same time period (no historical controls)
(11) Baseline equivalence of groups: The groups should be similar regarding the criteria other than the studied endpoints. Absence of confounding factors that could bias the interpretation of results
(12) Adequate statistical analyses: Whether the statistics were in accordance with the type of study with calculation of confidence intervals or relative risk.

From: Slim K, Nini E, Forestier D, Kwiatkowski F, Panis Y, Chipponi J. Methodological index for non-randomized studies (minors): development and validation of a new instrument. ANZ J Surg. 2003 Sep;73(9):712-6. doi: 10.1046/j.1445-2197.2003.02748.x. PMID: 12956787.

Modified JBI critical appraisal checklist for studies reporting prevalence data

The JBI Critical Appraisal Checklist for Studies Reporting Prevalence Data was adapted to suit the objectives of this review, as osteoradionecrosis (ORN) was reported as a secondary rather than primary outcome in most included studies. Specific items were modified to better capture aspects of outcome detection, reporting completeness, and the assessment of data reliability in this context. These adaptations were intended to account for the heterogeneity in ORN reporting and the limitations inherent to retrospective and non-prevalence-focused study designs.

The items of the original checklist related to the appropriateness of statistical analysis and response rate were omitted, as ORN was not analyzed as a primary outcome in the included studies.

The adapted checklist comprised 8 items, resulting in a maximum score of 8 points per study. For improved comparability and integration into the statistical analysis, scores were converted to a percentage scale by multiplying by 100.

All modifications are explicitly indicated in the itemized checklist below.

(1) Was the sample frame appropriate to address the target population?
Yes/no/unclear/not applicable
(2) Were study participants sampled in an appropriate way?
Yes/no/unclear/not applicable
(3) Was the sample size adequate?
Yes/no/unclear/not applicable
(4) Were the study subjects and the setting described in detail?
Yes/no/unclear/not applicable
(5) Were valid methods used for the identification of the condition?
Yes/no/unclear/not applicable
(6) Was the condition measured in a standard, reliable way for all participants?
Yes/no/unclear/not applicable
(7) ***Modified from original checklist****:* Was the follow-up duration adequate for detection?
Yes/no/unclear/not applicable
(8) ***Modified from original checklist***: Were all diagnosis related stages reported?
Yes/no/unclear/not applicable

From: Munn Z, Moola S, Lisy K, Riitano D, Tufanaru C. Methodological guidance for systematic reviews of observational epidemiological studies reporting prevalence and incidence data. Int J Evid Based Healthc. 2015;13(3):147–153.

Cochrane Risk of Bias 2 (RoB 2) tool

For the randomized controlled trial assessed using the Cochrane Risk of Bias 2 (RoB 2) tool, qualitative judgments (“low risk”, “some concerns”, and “high risk” of bias) were translated into a quantitative scale to allow integration into the statistical analysis.
Specifically, studies rated as “low risk” were assigned values up to 100%, those with “some concerns” up to 66%, and those with “high risk” up to 33%. This transformation was applied to enable comparability with the percentage-based quality scores derived from the MINORS and adapted JBI assessments.

1. Bias arising from the randomisation process
1.1 Was the allocation sequence random?
1.2 Was the allocation sequence concealed until participants were enrolled and assigned to interventions?
1.3 Did baseline differences between intervention groups suggest a problem with the randomisation process?
Risk-of-bias judgment (low/high/some concerns)

2. Bias due to deviations from intended interventions
2.1 Were participants aware of their assigned intervention during the trial?
2.2 Were carers and people delivering the interventions aware of participants assigned intervention during the trial?
2.3 If Y/PY/NI to 2.1 or 2.2: Were there deviations from the intended intervention that arose because of the trial context?
2.4 If Y/PY/NI to 2.3: Were these deviations likely to have affected the outcome?
2.5 If Y/PY to 2.4: Were these deviations from intended intervention balanced between groups?
2.6 Was an appropriate analysis used to estimate the effect of assignment to intervention?
2.7 If N/PN/NI to 2.6: Was there potential for a substantial impact (on the result) of the failure to analyse participants in the group to which they were randomised?
Risk-of-bias judgment (low/high/some concerns)

3. Bias due to missing outcome data
3.1 Were data for this outcome available for all, or nearly all, participants randomised?
3.2 If N/PN/NI to 3.1: Is there evidence that the result was not biased by missing outcome data?
3.3 If N/PN to 3.2: Could missingness in the outcome depend on its true value?
3.4 If Y/PY/NI to 3.3: Is it likely that missingness in the outcome depended on its true value?
Risk-of-bias judgment (low/high/some concerns)

4. Bias in measurement of the outcome
4.1 Was the method of measuring the outcome inappropriate?
4.2 Could measurement or ascertainment of the outcome have differed between intervention groups?
4.3 If N/PN/NI to 4.1 and 4.2: Were outcome assessors aware of the intervention received by study participants?
4.4 If Y/PY/NI to 4.3: Could assessment of the outcome have been influenced by knowledge of intervention received?
4.5 If Y/PY/NI to 4.4: Is it likely that assessment of the outcome was influenced by knowledge of intervention received?
Risk-of-bias judgment (low/high/some concerns)

5. Bias in selection of the reported results

5.1 Were the data that produced this result analysed in accordance with a prespecified analysis plan that was finalised before unblinded outcome data were available for analysis?
Is the numerical result being assessed likely to have been selected, on the basis of the results, from:
5.2 ... multiple eligible outcome measurements (eg, scales, definitions, time points) within the outcome domain?
5.3 ... multiple eligible analyses of the data?
Risk-of-bias judgment (low/high/some concerns)

Overall bias
Risk-of-bias judgment (low/high/some concerns)

From: Sterne JAC, Savović J, Page MJ, Elbers RG, Blencowe NS, Boutron I, Cates CJ, Cheng HY, Corbett MS, Eldridge SM, Emberson JR, Hernán MA, Hopewell S, Hróbjartsson A, Junqueira DR, Jüni P, Kirkham JJ, Lasserson T, Li T, McAleenan A, Reeves BC, Shepperd S, Shrier I, Stewart LA, Tilling K, White IR, Whiting PF, Higgins JPT. RoB 2: a revised tool for assessing risk of bias in randomised trials. BMJ. 2019 Aug 28;366:l4898. doi: 10.1136/bmj.l4898. PMID: 31462531.

Supplementary Material (G): Excludes studies with reason

Author, year	Title	Reason for exclusion
Grossman, C. E.; Mick, R.; Langer, C. J.; Lin, A.; Wan, F.; Zhang, Q.; Ang, K.; Spencer, S. A.; Horwitz, E. M.; Wong, S.; Le, Q.; Kanetsky, P. A., 2014	Prognostic Nomograms for Toxicity and Survival After Head and Neck Cancer Reirradiation and Chemotherapy: A Secondary Analysis of RTOG 9610 and 9911	No toxicity reported
Held, Thomas; Lang, Kristin; Regnery, Sebastian; Weusthof, Katharina; Hommertgen, Adriane; Jäkel, Cornelia; Tonndorf-Martini, Eric; Krisam, Johannes; Plinkert, Peter; Zaoui, Karim; Freudlsperger, Christian; Moratin, Julius; Krauss, Jürgen; Harrabi, Semi B.; Herfarth, Klaus; Debus, Jürgen; Adeberg, Sebastian; 2020	Carbon ion reirradiation compared to intensity-modulated re-radiotherapy for recurrent head and neck cancer (CARE): a randomized controlled trial	No toxicity reported
Jensen, A. D.; Nikoghosyan, A.; Ellerbrock, M.; Ecker, S.; Debus, J.; Münter, M. W.; 2011	Re-irradiation with scanned charged particle beams in recurrent tumours of the head and neck: Acute toxicity and feasibility	No toxicity reported
Li, You Quan; Tian, Yun Ming; Tan, Sze Huey; Liu, Ming Zhu; Kusumawidjaja, Grace; Ong, Enya H. W.; Zhao, Chong; Tan, Terence W. K.; Fong, Kam Weng; Sommat, Kiattisa; Soong, Yoke Lim; Wee, Joseph T. S.; Han, Fei; Chua, Melvin L. K.; 2018	Prognostic Model for Stratification of Radioresistant Nasopharynx Carcinoma to Curative Salvage Radiotherapy	No toxicity reported
May, M. E.; Cash, E. D.; Silverman, C. L.; Redman, R. A.; Perez, C. A.; Wilson, L. D.; Tennant, P. A.; Bumpous, J. M.; Dunlap, N. E.; 2019	Prognostic factors and selection criteria in the retreatment of head and neck cancers	No toxicity reported
Orecchia, R.; Redda, M. G.; Ragona, R.; Nassisi, D.; Jereczek-Fossa, B.; Zurrida, S.; Bussi, M.; Succo, G.; Sannazzari, G.; 1999	Results of hypofractionated stereotactic re-irradiation on 13 locally recurrent nasopharyngeal carcinomas	No toxicity reported
Schleicher, U. M.; Andreopoulos, D.; Ammon, J.; 2001	Palliative radiotherapy in recurrent head-and-neck tumors by a percutaneous superfractionated treatment schedule	No toxicity reported
Svajdova, Michaela; Sicak, Marian; Dubinsky, Pavol; Slavik, Marek; Slampa, Pavel; Kazda, Tomas; 2020	Recurrent Nasopharyngeal Cancer: Critical Review of Local Treatment Options Including Recommendations during the COVID-19 Pandemic	No toxicity reported
Velez, Maria A.; Wang, Pin-Chieh; Hsu, Sophia; Chin, Robert; Beron, Philip; Abemayor, Elliot; St John, Maie; Chen, Allen M.; 2018	Prognostic significance of HPV status in the re-irradiation of recurrent and second primary cancers of the head and neck	No toxicity reported
Yu, Kwok Hung; Leung, Sing Fai; Tung, Stewart Yuk; Zee, Benny; Chua, Daniel T. T.; Sze, Wai Man; Law, Stephen C. K.; Kam, Michael K. M.; Leung, To Wai; Sham, Jonathan S. T.; Lee, Anne W. M.; Au, Joseph S. K.; Hui, Edwin P.; Sze, Wing Kin; Cheng, Ashley C. K.; Yau, T. K.; Ngan, Roger K. C.; Wong, Frank C. S.; Au, Gordon K. H.; Chan, Anthony T. C.; 2005	Survival outcome of patients with nasopharyngeal carcinoma with first local failure: a study by the Hong Kong Nasopharyngeal Carcinoma Study Group	No toxicity reported
Cho, J. H.; Kim, G. E.; Cho, K. H.; Lee, C. G.; Kim, Y. B.; Lee, S. W.; Keum, K. C.; Suh, C. O.; 2001	Hyperfractionated re-irradiation using a 3-dimensional conformal technique for locally recurrent carcinoma of the nasopharynx; preliminary results	Case series without consecutive inclusion mentioned
Chua, D. T.; Sham, J. S.; Hung, K. N.; Kwong, D. L.; Kwong, P. W.; Leung, L. H.; 1999	Stereotactic radiosurgery as a salvage treatment for locally persistent and recurrent nasopharyngeal carcinoma	Case series without consecutive inclusion mentioned
Gondi, Suma; Reichl, Kaitlyn; Tassone, Patrick; 2024	Reirradiation after osteocutaneous free flap reconstruction for locally recurrent head and neck cancer	Case series without consecutive inclusion mentioned
Hazkani, I.; Rabinovics, N.; Limon, D.; Silvern, D.; Koren, S.; Hadar, T.; Bachar, G.; Shpitzer, T.; Popovtzer, A.; 2016	Brachytherapy for Radiotherapy-Resistant Head and Neck Cancer: A Review of a Single Center Experience	Case series without consecutive inclusion mentioned
Jeong, Songmi; Yoo, Eun Jung; Kim, Ji Yoon; Han, Chi Wha; Kim, Ki Jun; Kay, Chul Seung; 2013	Re-irradiation of unresectable recurrent head and neck cancer: using Helical	Case series without consecutive inclusion mentioned

	Tomotherapy as image-guided intensity-modulated radiotherapy	
Konishi, Masaru; Takeuchi, Yuki; Kubo, Katsumaro; Imano, Nobuki; Nishibuchi, Ikuno; Murakami, Yuji; Shimabukuro, Kiichi; Wongratwanich, Pongsapak; Kakimoto, Naoya; Nagata, Yasushi; 2022	Reirradiation Using (198)Au Grain Brachytherapy for Recurrent Oral Cancer Cases Previously Treated by Definitive Radiotherapy	Case series without consecutive inclusion mentioned
Krempien, Robert C.; Grehn, Christian; Haag, Claudia; Straulino, Anika; Hensley, Frank W.; Kotrikova, Bibiana; Hofele, Christof; Debus, Juergen; Harms, Wolfgang; 2005	Feasibility report for retreatment of locally recurrent head-and-neck cancers by combined brachy-chemotherapy using frameless image-guided 3D interstitial brachytherapy	Case series without consecutive inclusion mentioned
Pommier, P.; Bolot, G.; Martel, I.; Montbarbon, X.; Ardiet, J. M.; Coquart, R.; Poupart, M.; Leval, J.; Ramade, A.; Dubreuilh, C.; Pignat, J. C.; Carrie, C.; 1997	Salvage brachytherapy of posterior pharyngeal wall squamous cell carcinoma in a previously irradiated area	Case series without consecutive inclusion mentioned
Rades, Dirk; Seidl, Daniel; Wollenberg, Barbara; Schild, Steven E.; Hakim, Samer G.; 2016	Radiochemotherapy with Paclitaxel for Recurrent Previously Irradiated Squamous Cell Carcinoma of the Head and Neck	Case series without consecutive inclusion mentioned
Schiefke, F.; Hildebrandt, G.; Pohlmann, S.; Heinicke, F.; Hemprich, A.; Frerich, B.; 2008	Combination of surgical resection and HDR-brachytherapy in patients with recurrent or advanced head and neck carcinomas	Case series without consecutive inclusion mentioned
Tan, E. H.; Adelstein, D. J.; Saxton, J. P.; Wood, B. G.; Eliachar, I.; van Kirk, M. A.; Lavertu, P.; 1997	Concurrent chemoradiotherapy for salvage in relapsed squamous cell head and neck cancer	Case series without consecutive inclusion mentioned
Teudt, Ingo U.; Kovàcs, György; Ritter, Matthias; Melchert, Corinna; Soror, Tamer; Wollenberg, Barbara; Meyer, Jens E.; 2016	Intensity modulated perioperative HDR brachytherapy for recurrent and/or advanced head and neck metastases	Case series without consecutive inclusion mentioned
van Waes, Carter; Chang, Angela A.; Lebowitz, Peter F.; Druzgal, Colleen H.; Chen, Zhong; Elsayed, Yusri A.; Sunwoo, John B.; Rudy, Susan F.; Morris, John C.; Mitchell, James B.; Camphausen, Kevin; Gius, David; Adams, Julian; Sausville, Edward A.; Conley, Barbara A.; 2005	Inhibition of nuclear factor-kappaB and target genes during combined therapy with proteasome inhibitor bortezomib and reirradiation in patients with recurrent head-and-neck squamous cell carcinoma	Case series without consecutive inclusion mentioned
Zwicker, Felix; Roeder, Falk; Thieke, Christian; Timke, Carmen; Münter, Marc W.; Huber, Peter E.; Debus, Jürgen; 2001	IMRT reirradiation with concurrent cetuximab immunotherapy in recurrent head and neck cancer	Case series without consecutive inclusion mentioned
Modesto, A.; Filleron, T.; Chevreau, C.; Le Pechoux, C.; Rochaix, P.; Le Guellec, S.; Ducassou, A.; Gangloff, D.; Ferron, G.; Delannes, M.; 2014	Role of radiation therapy in the conservative management of sarcoma within an irradiated field	Other cancer than HNC involved
Ansinelli, Hayden; Singh, Raj; Sharma, Dana L.; Jenkins, Jan; Davis, Joanne; Vargo, John A.; Sharma, Sanjeev; 2018	Salvage Stereotactic Body Radiation Therapy for Locally Recurrent Previously Irradiated Head and Neck Squamous Cell Carcinoma: An Analysis from the RSSearch® Registry	Overlapping of patients' population
Balermpas, P.; Hambek, M.; Seitz, O.; Rödel, C.; Weiss, C.; 2009	Combined Cetuximab and Reirradiation for Locoregional Recurrent and Inoperable Squamous Cell Carcinoma of the Head and Neck	Overlapping of patients' population
Balermpas, P.; Keller, C.; Hambek, M.; Wagenblast, J.; Seitz, O.; Rödel, C.; Weiss, C.; 2012	Reirradiation With Cetuximab in Locoregional Recurrent and Inoperable Squamous Cell Carcinoma of the Head and Neck: Feasibility and First Efficacy Results	Overlapping of patients' population
Beddok, Arnaud; Saint-Martin, Caroline; Krhili, Samar; Eddine, Catherine Ala; Champion, Laurence; Chilles, Anne; Goudjil, Farid; Zefkili, Sofia; Amessis, Malika; Peurien, Dominique; Choussy, Olivier; Le Tourneau, Christophe; Dendale, Remi; Buvat, Irene; Créhange, Gilles; Calugaru, Valentin; 2022	Curative high-dose reirradiation for patients with recurrent head and neck squamous cell carcinoma using IMRT or proton therapy: Outcomes and analysis of patterns of failure	Overlapping of patients' population
Gao, Jing; Hu, Jiyi; Guan, Xiyin; Yang, Jing; Hu, Weixu; Kong, Lin; Lu, Jiade J.; 2019	Salvage Carbon-Ion Radiation Therapy For Locoregionally Recurrent Head and Neck Malignancies	Overlapping of patients' population
Hayashi, Y.; Nakamura, T.; Mitsudo, K.; Yamaguchi, H.; Ono, T.; Azami, Y.; Takayama, K.; Suzuki, M.; Hatayama, Y.; Tsukiyama, I.; Hareyama, M.; Kikuchi, Y.; Fuwa, N.; Tohnai, I.; 2016	Retrograde intra-arterial chemotherapy and daily concurrent proton beam therapy for recurrent oral cavity squamous cell carcinoma: Analysis of therapeutic results in 46 cases	Overlapping of patients' population
Hehr, Thomas; Classen, Johannes; Belka, Claus; Welz, Stefan; Schäfer, Juergen;	Reirradiation alternating with docetaxel and cisplatin in inoperable recurrence of	Overlapping of patients' population

Koitschev, Assen; Bamberg, Michael; Budach, Wilfried; 2005	head-and-neck cancer: a prospective phase I/II trial	
Hu, Jiyi; Bao, Cihang; Gao, Jing; Guan, Xiyin; Hu, Weixu; Yang, Jing; Hu, Chaosu; Kong, Lin; Lu, Jiade J.; 2018	Salvage treatment using carbon ion radiation in patients with locoregionally recurrent nasopharyngeal carcinoma: Initial results	Overlapping of patients' population
Kodani, Naohiro; Yamazaki, Hideya; Tsubokura, Takuji; Shiomi, Hiroya; Kobayashi, Kana; Nishimura, Takuya; Aibe, Norihiro; Ikeno, Hiroyasu; Nishimura, Tsunehiko; 2011	Stereotactic body radiation therapy for head and neck tumor: disease control and morbidity outcomes	Overlapping of patients' population
Martínez-Monge, R.; Divassón, M. P.; Cambeiro, M.; Gaztañaga, M.; Moreno, M.; Arbea, L.; Montesdeoca, N.; Alcalde, J.; 2011	DETERMINANTS OF COMPLICATIONS AND OUTCOME IN HIGH-RISK SQUAMOUS CELL HEAD-AND-NECK CANCER TREATED WITH PERIOPERATIVE HIGH-DOSE RATE BRACHYTHERAPY (PHDRB)	Overlapping of patients' population
Milanović, D.; Jeremić, B.; Grosu, A. L.; Rücker, G.; Henke, M.; 2013	Reirradiation plus EGFR inhibition in locally recurrent and unresectable head and neck cancer: final results from a single institution	Overlapping of patients' population
Popovtzer, Aron; Gluck, Iris; Chepeha, Douglas B.; Teknos, Theodoros N.; Moyer, Jeffrey S.; Prince, Mark E.; Bradford, Carol R.; Eisbruch, Avraham; 2009	The pattern of failure after reirradiation of recurrent squamous cell head and neck cancer: implications for defining the targets	Overlapping of patients' population
Rudzianskas, V.; Inciura, A.; Juozaityte, E.; Rudzianskiene, M.; Kubilius, R.; Vaitkus, S.; Kaseta, M.; Adliene, D.; 2012	Reirradiation of recurrent head and neck cancer using high-dose-rate brachytherapy	Overlapping of patients' population
Rwigema, Jean-Claude; Heron, Dwight E.; Ferris, Robert L.; Gibson, Michael; Quinn, Annette; Yang, Yong; Ozhasoglu, Cihat; Burton, Steven; 2010	Fractionated stereotactic body radiation therapy in the treatment of previously-irradiated recurrent head and neck carcinoma: updated report of the University of Pittsburgh experience	Overlapping of patients' population
Spencer, S. A.; Harris, J.; Wheeler, R. H.; Machtay, M.; Schultz, C.; Spanos, W.; Rotman, M.; Meredith, R.; 2001	RTOG 96-10: reirradiation with concurrent hydroxyurea and 5-fluorouracil in patients with squamous cell cancer of the head and neck	Overlapping of patients' population
Spencer, S. A.; Wheeler, R. H.; Peters, G. E.; Beenken, S. W.; Meredith, R. F.; Smith, J.; Conner, W.; Salter, M. M.; 1999	Concomitant chemotherapy and reirradiation as management for recurrent cancer of the head and neck	Overlapping of patients' population
Strnad, V.; Lotter, M.; Kreppner, S.; Fietkau, R.; 2014	Re-irradiation with interstitial pulsed-dose-rate brachytherapy for unresectable recurrent head and neck carcinoma	Overlapping of patients' population
Tian, Y-M; Tian, Y-H; Zeng, L.; Liu, S.; Guan, Y.; Lu, T-X; Han, F.; 2014	Prognostic model for survival of local recurrent nasopharyngeal carcinoma with intensity-modulated radiotherapy	Overlapping of patients' population
Vargo, J. A.; Wegner, R. E.; Heron, de; Ferris, R. L.; Rwigema, J. C.M.; Quinn, A.; Gigliotti, P.; Ohr, J.; Kubicek, G. J.; Burton, S.; 2012	Stereotactic body radiation therapy for locally recurrent, previously irradiated nonsquamous cell cancers of the head and neck	Overlapping of patients' population
Ward, Matthew C.; Lee, Nancy Y.; Caudell, Jimmy J.; Zajichek, Alexander; Awan, Musaddiq J.; Koyfman, Shlomo A.; Dunlap, Neal E.; Zakem, Sara J.; Hassanzadeh, Comron; Marcrom, Samuel; Boggs, Drexell H.; Isrow, Derek; Vargo, John A.; Heron, Dwight E.; Siddiqui, Farzan; Bonner, James A.; Beitler, Jonathan J.; Yao, Min; Trotti, Andy M.; Riaz, Nadeem; 2019	A competing risk nomogram to predict severe late toxicity after modern re-irradiation for squamous carcinoma of the head and neck	Overlapping of patients' population
Yamazaki, H.; Ogita, M.; Himei, K.; Nakamura, S.; Suzuki, G.; Yoshida, K.; Kotsuma, T.; Yoshioka, Y.; 2016	Reirradiation using robotic image-guided stereotactic radiotherapy of recurrent head and neck cancer	Overlapping of patients' population
Yamazaki, Hideya; Ogita, Mikio; Himei, Kengo; Nakamura, Satoaki; Suzuki, Gen; Kotsuma, Tadayuki; Yoshida, Ken; Yoshioka, Yasuo; 2017	Effect of intratumoral abscess/necrosis on the outcome for head and neck cancer patients treated by hypofractionated stereotactic re-irradiation using CyberKnife®	Overlapping of patients' population
Dogan, Ozlem Yetmen; Yaprak, Gokhan; Ozyurt, Hazan; Bicakci, Beyhan Ceylaner; Demircioglu, Fatih; Algul, Emriye; Isik, Naciye; 2022	Is fractionated robotic stereotactic body radiosurgery optional salvage treatment for the re-irradiation of locally recurrent nasopharyngeal carcinoma?	Retraction
Ohizumi, Y.; Tamai, Y.; Imamiya, S.; Akiba, T.; 2002	Complications following re-irradiation for head and neck cancer	Necrosis excluded
Ohizumi, Yukio; Tamai, Yoshifumi; Imamiya, Satoshi; Akiba, Takeshi; 2002	Prognostic factors of reirradiation for recurrent head and neck cancer	Necrosis excluded

Lee, D. S.; Kim, Y. S.; Cheon, J. S.; Song, J. H.; Son, S. H.; Jang, J. S.; Kang, Y. N.; Kang, J. H.; Jung, S. L.; Yoo, I. R.; Jang, H. S.; 2012	Long-term outcome and toxicity of hypofractionated stereotactic body radiotherapy as a boost treatment for head and neck cancer: the importance of boost volume assessment	No Reirradiation
Martínez-Monge, Rafael; Gómez-Iturriaga, Alfonso; Cambeiro, Mauricio; Garrán, Cristina; Montesdeoca, Néstor; Aristu, José Javier; Alcalde, Juan; 2009	Phase I-II trial of perioperative high-dose-rate brachytherapy in oral cavity and oropharyngeal cancer	No Reirradiation
Claus, Filip; Duthoy, Wim; Boterberg, Tom; Gersem, Werner de; Huys, John; Vermeersch, Hubert; Neve, Wilfried de; 2002	Intensity modulated radiation therapy for oropharyngeal and oral cavity tumors: clinical use and experience	Mixed cohorts
Glatzel, Michael; Büntzel, Jens; Schröder, Dirk; Küttner, Klaus; Fröhlich, Dietmar; 2002	High-dose-rate brachytherapy in the treatment of recurrent and residual head and neck cancer	Mixed cohorts
Gunn, G. B.; Garden, A. S.; Ye, R.; Ausat, N.; Dahlstrom, K. R.; Morrison, W. H.; Fuller, C. D.; Phan, J.; Reddy, J. P.; Shah, S. J.; Mayo, L. L.; Chun, S. G.; Chronowski, G. M.; Moreno, A. C.; Myers, J. N.; Hanna, E. Y.; Esmaeli, B.; Gillison, M. L.; Ferrarotto, R.; Hutcheson, K. A.; Chambers, M. S.; Le Ginsberg; El-Naggar, A. K.; Di Rosenthal; Zhu, X. R.R.; Frank, S. J.; 2021	Proton Therapy for Head and Neck Cancer: A 12-Year, Single-Institution Experience	Mixed cohorts
Jensen, Alexandra D.; Nikoghosyan, Anna V.; Ecker, Swantje; Ellerbrock, Malte; Debus, Jürgen; Münter, Marc W.; 2011	Carbon ion therapy for advanced sinonasal malignancies: feasibility and acute toxicity	Mixed cohorts
Narayana, A.; Cohen, G. N.; Zaider, M.; Chan, K.; Lee, N.; Wong, R. J.; Boyle, J.; Shaha, A.; Kraus, D.; Shah, J.; Zelefsky, M. J.; 2007	High-dose-rate interstitial brachytherapy in recurrent and previously irradiated head and neck cancers - Preliminary results	Mixed cohorts
Ozyigit, Gokhan; Cengiz, Mustafa; Hurmuz, Pervin; Yazici, Gozde; Gultekin, Melis; Akyol, Fadil; Yildiz, Ferah; Gurkaynak, Murat; Zorlu, Faruk; 2014	Robotic stereotactic radiosurgery in patients with nasal cavity and paranasal sinus tumors	Mixed cohorts
Pham, Anthony; Arora, Shruthi; Wernicke, A. Gabriella; Kutler, David I.; Cohen, Marc; Kuhel, William; Trichter, Samuel; Nori, Dattatreyudu; Formenti, Silvia C.; Parashar, Bhupesh; 2015	Cesium-131 brachytherapy in high risk and recurrent head and neck cancers: first report of long-term outcomes	Mixed cohorts
Soror, T.; Paul, J.; Melchert, C.; Idel, C.; Rades, D.; Bruchhage, K. L.; Kovács, G.; Leichtle, A.; 2023	Salvage High-Dose-Rate Interventional Radiotherapy (Brachytherapy) Combined with Surgery for Regionally Relapsed Head and Neck Cancers	Mixed cohorts
Teckie, Sewit; Lok, Benjamin H.; Rao, Shyam; Gutiontov, Stanley I.; Yamada, Yoshiya; Berry, Sean L.; Zelefsky, Michael J.; Lee, Nancy Y.; 2016	High-dose hypofractionated radiotherapy is effective and safe for tumors in the head-and-neck	Mixed cohorts
Ahlawat, Parveen; Rawat, Sheh; Kakria, Anjali; Devnani, Bharti; Wahi, Inderjit Kaur; Simson, David K.; 2017	Reirradiation with IMRT for recurrent head and neck cancer: A single-institutional report on disease control, survival, and toxicity	ORN without information about localisation
Altay-Langguth, Alev; Balermpas, Panagiotis; Brandts, Christian; Balster, Sven; Ghanaati, Shahram; Winkelmann, Ria; Burck, Iris; Rödel, Franz; Martin, Daniel; Rödel, Claus; Grün, Jens von der; 2021	Re-irradiation with concurrent and maintenance nivolumab in locally recurrent and inoperable squamous cell carcinoma of the head and neck: A single-center cohort study	ORN without information about localisation
Amdal, Cecilie Delphin; Moan, Jon Magne; Dale, Einar; Falk, Ragnhild Sørum; Johansen, Safora; Bjordal, Kristin; 2023	Impact of hyperfractionated re-irradiation on quality of life in patients with recurrent or second primary head and neck cancer, a prospective single institutional study	ORN without information about localisation
Bagley, af; Garden, A. S.; Reddy, J. P.; Moreno, A. C.; Frank, S. J.; Di Rosenthal; Morrison, W. H.; Gunn, G. B.; Fuller, C. D.; Shah, S. J.; Ferrarotto, R.; Sturgis, E. M.; Gross, N. D.; Phan, J.; 2020	Highly conformalreirradiation in patients with prior oropharyngeal radiation: Clinical efficacy and toxicity outcomes	ORN without information about localisation
Breen, William; Kelly, Jacqueline; Park, Henry S.; Son, Yung; Sasaki, Clarence; Wilson, Lynn; Decker, Roy; Husain, Zain A.; 2018	Permanent Interstitial Brachytherapy for Previously Irradiated Head and Neck Cancer	ORN without information about localisation
Caudell, J. J.; Ward, M. C.; Riaz, N.; Zakem, S. J.; Awan, M. J.; Dunlap, N. E.; Isrow, D.; Hassanzadeh, C.; Vargo, J. A.; Heron, de; Marcrom, S.; Boggs, D. H.; Reddy, C. A.; Dault, J.; Bonner, J. A.;	Volume, Dose, and Fractionation Considerations for IMRT-based Reirradiation in Head and Neck Cancer: A Multi-institution Analysis	ORN without information about localisation

Higgins, K. A.; Beitler, J. J.; Koyfman, S. A.; Machtay, M.; Yao, M.; Trotti, am; Siddiqui, F.; Lee, N. Y.; MIRI Collaborative; 2018		
Chen, Allen M.; Harris, Jeremy P.; Nabar, Rupali; Tjoa, Tjoson; Haidar, Yarah; Armstrong, William B.; 2024	Re-irradiation versus systemic therapy for the management of local-regionally recurrent head and neck cancer	ORN without information about localisation
Chen, Yen-Chao; Fan, Kang-Hsing; Lin, Chien-Yu; Kang, Chung-Jan; Huang, Shiang-Fu; Wang, Hung-Ming; Cheng, Ann-Joy; Chang, Joseph Tung-Chieh; 2022	Outcomes of re-irradiation for oral cavity squamous cell carcinoma	ORN without information about localisation
Choe, Kevin S.; Haraf, Daniel J.; Solanki, Abhishek; Cohen, Ezra E. W.; Seiwert, Tanguy Y.; Stenson, Kerstin M.; Blair, Elizabeth A.; Portugal, Louis; Villaflor, Victoria M.; Witt, Mary Ellyn; Vokes, Everett E.; Salama, Joseph K.; 2011	Prior chemoradiotherapy adversely impacts outcomes of recurrent and second primary head and neck cancer treated with concurrent chemotherapy and reirradiation	ORN without information about localisation
Chua, Daniel T. T.; Sham, Jonathan S. T.; Hung, Kwan-Ngai; Leung, Lucullus H. T.; Au, Gordon K. H.; 2006	Predictive factors of tumor control and survival after radiosurgery for local failures of nasopharyngeal carcinoma	ORN without information about localisation
Crevoisier, R. de; Bourhis, J.; Domenge, C.; WIBAULT, P.; Koscielny, S.; Lusinchi, A.; Mamelle, G.; Janot, F.; Julieron, M.; Leridant, A. M.; MARANDAS, P.; ARMAND, J. P.; Schwaab, G.; LUBOINSKI, B.; Eschwege, F.; 1998	Full-dose reirradiation for unresectable head and neck carcinoma: experience at the Gustave-Roussy Institute in a series of 169 patients	ORN without information about localisation
Curtis, Kelly K.; Ross, Helen J.; Garrett, Ashley L.; Jizba, Theresa A.; Patel, Ajay B.; Patel, Samir H.; Wong, William W.; Halyard, Michele Y.; Ko, Stephen J.; Kosiorek, Heidi E.; Foote, Robert L.; 2016	Outcomes of patients with loco-regionally recurrent or new primary squamous cell carcinomas of the head and neck treated with curative intent reirradiation at Mayo Clinic	ORN without information about localisation
Dawson, L. A.; Myers, L. L.; Bradford, C. R.; Chepeha, D. B.; Hogikyan, N. D.; Teknos, T. N.; Terrell, J. E.; Wolf, G. T.; Eisbruch, A.; 2001	Conformal re-irradiation of recurrent and new primary head-and-neck cancer	ORN without information about localisation
Delerue, Chloé; Pasquier, David; Bogart, Emilie; Mirabel, Xavier; Laffarguette, Julien; Lals, Séverine; Barthoulot, Maël; Lartigau, Eric; Liem, Xavier; 2024	Stereotactic reirradiation in the treatment of head and neck cancers: A retrospective study on the long-term experience of the Oscar Lambret Center	ORN without information about localisation
Diao, K.; Nguyen, T. P.; Moreno, A. C.; Reddy, J. P.; Garden, A. S.; Wang, C. H.; Tung, S.; Wang, C. J.; Wang, X. A.; Di Rosenthal; Fuller, C. D.; Gunn, G. B.; Frank, S. J.; Morrison, W. H.; Shah, S. J.; Lee, A.; Spiotto, M. T.; Su, S. Y.; Ferrarotto, R.; Phan, J.; 2021	Stereotactic body ablative radiotherapy for reirradiation of small volume head and neck cancers is associated with prolonged survival: A large, single-institution, modern cohort study	ORN without information about localisation
Doi, Hiroshi; Uemoto, Kenji; Masai, Norihisa; Tatsumi, Daisaku; Shiomi, Hiroya; Oh, Ryoong-Jin; 2018	Definitive re-irradiation using intensity-modulated radiation therapy in cancers of the head and neck, focusing on rare tumors	ORN without information about localisation
Duprez, F.; Berwouts, D.; Madani, I.; Bonte, K.; Boterberg, T.; Gersem, W. de; Deron, P.; Huvenne, W.; Neve, W. de; 2014	High-dose reirradiation with intensity-modulated radiotherapy for recurrent head-and-neck cancer: Disease control, survival and toxicity	ORN without information about localisation
Fan, Ming; Kang, Jung Julie; Lee, Anna; Fan, Dan; Wang, Huili; Kitpanit, Sarin; Fox, Pamela; Sine, Kevin; Mah, Dennis; McBride, Sean M.; Tsai, Chiaojung Jillian; Riaz, Nadeem; Dunn, Lara A.; Sherman, Eric J.; Michel, Loren; Singh, Bhuvanesh; Ganly, Ian; Wong, Richard J.; Boyle, Jay O.; Cohen, Marc A.; Lee, Nancy Y.; 2020	Outcomes and toxicities of definitive radiotherapy and reirradiation using 3-dimensional conformal or intensity-modulated (pencil beam) proton therapy for patients with nasal cavity and paranasal sinus malignancies	ORN without information about localisation
Gogineni, E.; Zhang, I.; Rana, Z.; Marrero, M.; Gill, G.; Sharma, A.; Riegel, A. C.; Teckie, S.; Ghaly, M.; 2019	Quality of Life Outcomes Following Organ-Sparing SBRT in Previously Irradiated Recurrent Head and Neck Cancer	ORN without information about localisation
Hayashi, Kazuhiko; Koto, Masashi; Ikawa, Hiroaki; Hagiwara, Yasuhito; Tsuji, Hiroshi; Ogawa, Kazuhiko; Kamada, Tadashi; 2019	Feasibility of Re-irradiation using carbon ions for recurrent head and neck malignancies after carbon-ion radiotherapy	ORN without information about localisation
Hayashi, Yuichiro; Nakamura, Tatsuya; Mitsudo, Kenji; Kimura, Kanako; Yamaguchi, Hisashi; Ono, Takashi; Azami, Yusuke; Takayama, Kanako; Hirose, Katsumi; Yabuuchi, Tomonori; Suzuki, Motohisa; Hatayama, Yoshiomi; Kikuchi, Yasuhiro; Wada, Hitoshi; Fuwa,	Re-irradiation using proton beam therapy combined with weekly intra-arterial chemotherapy for recurrent oral cancer	ORN without information about localisation

Nobukazu; Hareyama, Masato; Tohnai, Iwai; 2017		
Held, Thomas; Harrabi, Semi B.; Lang, Kristin; Akbaba, Sati; Windisch, Paul; Bernhardt, Denise; Rieken, Stefan; Herfarth, Klaus; Debus, Jürgen; Adeberg, Sebastian; 2019	Dose-Limiting Organs at Risk in Carbon Ion Re-Irradiation of Head and Neck Malignancies: An Individual Risk-Benefit Tradeoff	ORN without information about localisation
Ho, Allen S.; Zumsteg, Zachary S.; Meyer, Annika; Riaz, Nadeem; Rahmati, Rahmatullah; Kraus, Dennis H.; McCarthy, Colleen; Wong, Richard J.; Shah, Jatin P.; Lee, Nancy Y.; 2016	Impact of Flap Reconstruction on Radiotoxicity After Salvage Surgery and Reirradiation for Recurrent Head and Neck Cancer	ORN without information about localisation
Hoebers, Frank; Heemsbergen, Wilma; Moor, Suzanne; Lopez, Marta; Klop, Martin; Tesselaar, Margot; Rasch, Coen; 2011	Reirradiation for head-and-neck cancer: delicate balance between effectiveness and toxicity	ORN without information about localisation
Iwata, Hiromitsu; Tatewaki, Koshi; Inoue, Mitsuhiro; Yokota, Naoki; Sato, Kengo; Shibamoto, Yuta; 2012	Salvage stereotactic reirradiation using the CyberKnife for the local recurrence of nasal or paranasal carcinoma	ORN without information about localisation
Janot, F.; Raucourt, D. de; Benhamou, E.; Ferron, C.; Dolivet, G.; Bensadoun, R. J.; Hamoir, M.; Géry, B.; Julieron, M.; Castaing, M.; Bardet, E.; Grégoire, V.; Bourhis, J.; 2008	Randomized Trial of Postoperative Reirradiation Combined With Chemotherapy After Salvage Surgery Compared With Salvage Surgery Alone in Head and Neck Carcinoma	ORN without information about localisation
Janssen, S.; Baumgartner, M.; Bremer, M.; Warszawski, A.; Stieve, M.; Eckardt, A.; Karstens, J-H; Meyer, A.; 2010	Re-irradiation of head and neck cancer-impact of total dose on outcome	ORN without information about localisation
Jensen, Alexandra D.; Poulakis, Melanie; Nikoghosyan, Anna V.; Chaudhri, Naved; Uhl, Matthias; Münter, Marc W.; Herfarth, Klaus K.; Debus, Jürgen; 2015	Re-irradiation of adenoid cystic carcinoma: analysis and evaluation of outcome in 52 consecutive patients treated with raster-scanned carbon ion therapy	ORN without information about localisation
Kawaguchi, Koji; Sato, Kengo; Horie, Akihisa; Iketani, Susumu; Yamada, Hiroyuki; Nakatani, Yasunori; Sato, Junichi; Hamada, Yoshiki; 2010	Stereotactic radiosurgery may contribute to overall survival for patients with recurrent head and neck carcinoma	ORN without information about localisation
Kharofa, J.; Choong, N.; Da Wang; Firat, S.; Schultz, C.; Sadasiwan, C.; Wong, S.; 2012	Continuous-Course Reirradiation With Concurrent Carboplatin and Paclitaxel for Locally Recurrent, Nonmetastatic Squamous Cell Carcinoma of the Head-and-Neck	ORN without information about localisation
Krc, Rebecca; Mendes, William; Molitoris, Jason; Ferris, Matthew; Song, Yang; Shetty, Amol; Mehra, Ranee; Papadimitriou, John C.; Hatten, Kyle; Taylor, Rodney; Wolf, Jeffrey; Sun, Kai; Bentzen, Soren; Regine, William; Tran, Phuoc; Witek, Matthew; 2024	Proton therapy re-irradiation outcomes and genomic landscape of patients with recurrent head and neck cancer	ORN without information about localisation
Kroesen, Michiel; van Holthe, Netteke; Sumser, Kemal; Chitu, Dana; Vernhout, Rene; Verduijn, Gerda; Franckena, Martine; Hardillo, Jose; van Rhoon, Gerard; Paulides, Margarethus; 2021	Feasibility, SAR Distribution, and Clinical Outcome upon Reirradiation and Deep Hyperthermia Using the Hypercollar3D in Head and Neck Cancer Patients	ORN without information about localisation
Lee, Anna; Woods, Robbie; Mahfouz, Amgad; Kitpanit, Sarin; Cartano, Olivia; Mohamed, Nader; Youssef, Irini; Marqueen, Kathryn; Sine, Kevin; Mah, Dennis; Neal, Brian; Zakeri, Kaveh; Kang, Jung J.; Riaz, Nadeem; Yu, Yao; McBride, Sean M.; Chen, Linda D.; Tsai, C. Jillian; Gelblum, Daphna Y.; Press, Robert H.; Michel, Loren S.; Sherman, Eric J.; Pfister, David; Dunn, Lara A.; Ho, Alan L.; Fetten, James; Wong, Richard J.; Boyle, Jay O.; Singh, Bhuvanesh; Cracchiolo, Jennifer R.; Ganly, Ian; Cohen, Marc A.; Lee, Nancy Y.; 2023	Evaluation of Proton Therapy Reirradiation for Patients With Recurrent Head and Neck Squamous Cell Carcinoma	ORN without information about localisation
Lee, H. I.; Kim, J. H.; Ahn, S. H.; Chung, E. J.; Keam, B.; Eom, K. Y.; Jeong, W. J.; Kim, J. W.; Wee, C. W.; Wu, H. G.; 2021	Re-irradiation for recurrent or second primary head and neck cancer	ORN without information about localisation
Lee, Jeongshim; Kim, Tae Hyung; Kim, Yeon-Sil; Kim, Myungsoo; Park, Jae Won; Kim, Sung Hyun; Kim, Hyun Ju; Lee, Chang Geol; 2020	Intensity-Modulated Radiotherapy-Based Reirradiation for Head and Neck Cancer: A Multi-institutional Study by Korean Radiation Oncology Group (KROG 1707)	ORN without information about localisation
Lee HI, Kim JH, Ahn SH, Chung EJ, Keam B, Eom KY, Jeong WJ, Kim JW, Wee CW, Wu HG.; 2016	Risk of Severe Toxicity According to Site of Recurrence in Patients Treated With Stereotactic Body Radiation Therapy for Recurrent Head and Neck Cancer	ORN without information about localisation

Liu, You-Ping; Wen, Yi-Hui; Tang, Jun; Wei, Yi; You, Rui; Zhu, Xiao-Lin; Li, Jian; Chen, Lin; Ling, Li; Zhang, Ning; Zou, Xiong; Hua, Yi-Jun; Chen, You-Mou; Chen, Lei; Lu, Li-Xia; Chen, Ming-Yuan; Wen, Wei-Ping; 2021	Endoscopic surgery compared with intensity-modulated radiotherapy in resectable locally recurrent nasopharyngeal carcinoma: a multicentre, open-label, randomised, controlled, phase 3 trial	ORN without information about localisation
Luginbuhl, Adam; Calder, Alyssa; Kutler, David; Zender, Chad; Wise-Draper, Trisha; Patel, Jena; Cheng, Michael; Karivedu, Vidhya; Zhan, Tingting; Parashar, Bhupesh; Gulati, Shuchi; Yao, Min; Lavertu, Pierre; Takiar, Vinita; Tang, Alice; Johnson, Jennifer; Keane, William; Curry, Joseph; Cognetti, David; Bar-Ad, Voichita; 2021	Multi-Institutional Study Validates Safety of Intraoperative Cesium-131 Brachytherapy for Treatment of Recurrent Head and Neck Cancer	ORN without information about localisation
Martínez-Monge, Rafael; Alcalde, Juan; Concejo, Carlos; Cambeiro, Mauricio; Garrán, Cristina; 2006	Perioperative high-dose-rate brachytherapy (PHDRB) in previously irradiated head and neck cancer: Initial results of a Phase I/II reirradiation study	ORN without information about localisation
Milano, Michael T.; Vokes, Everett E.; Salama, Joseph K.; Stenson, Kerstin M.; Kao, Johnny; Witt, Mary-Ellyn; Mittal, Bharat B.; Argiris, Athanassios; Weichselbaum, Ralph R.; Haraf, Daniel J.; 2005	Twice-daily reirradiation for recurrent and second primary head-and-neck cancer with gemcitabine, paclitaxel, and 5-fluorouracil chemotherapy	ORN without information about localisation
Mohamad, Issa; Abu Hejleh, Taher; Abdelqader, Sania; Wahbeh, Lina; Taqash, Ayat; Almousa, Abdelatif; Mayta, Ebrahim; Al-Ibraheem, Akram; Abuhijla, Fawzi; Abu-Hijlih, Ramiz; Hussein, Tariq; Al-Gargaz, Wisam; Ghatasheh, Hamza; Hosni, Ali; 2023	Re-Irradiation for Recurrent Head and Neck Cancer: Freedom from Cancer Recurrence Rate	ORN without information about localisation
Nam, Heerim; Ahn, Yong Chan; Yang, Kyungmi; Oh, Dongryul; Noh, Jae Myoung; 2022	Re-irradiation with Moderate Hypo-fractionation Using Intensity Modulated Photon or Proton Radiation Therapy in Locally Recurrent Squamous Cell Carcinoma of Nasopharynx	ORN without information about localisation
Ohnleiter, Thomas; Antoni, Delphine; Lefebvre, François; Truntzer, Pierre; Schultz, Philippe; Burgy, Mickael; Borel, Christian; Noël, Georges; 2018	Factors improving the outcome of patients re-irradiated with intensity-modulated radiotherapy (IMRT) for relapse or new head and neck cancer developed in irradiated areas	ORN without information about localisation
Phan, Jack; Sio, Terence T.; Nguyen, Theresa P.; Takiar, Vinita; Gunn, G. Brandon; Garden, Adam S.; Rosenthal, David I.; Fuller, Clifton D.; Morrison, William H.; Beadle, Beth; Ma, Dominic; Zafereo, Mark E.; Hutcheson, Kate A.; Kupferman, Michael E.; William, William N.; Frank, Steven J.; 2016	Reirradiation of Head and Neck Cancers With Proton Therapy: Outcomes and Analyses	ORN without information about localisation
Platteaux, N.; Dirix, P.; Vanstraelen, B.; Nuyts, S.; 2011	Outcome after Re-Irradiation of Head and Neck Cancer Patients	ORN without information about localisation
Puthawala, A.; Nisar Syed, A. M.; Gamie, S.; Chen, Y. J.; Londrc, A.; Nixon, V.; 2001	Interstitial low-dose-rate brachytherapy as a salvage treatment for recurrent head-and-neck cancers: long-term results	ORN without information about localisation
Riaz, Nadeem; Hong, Julian C.; Sherman, Eric J.; Morris, Luc; Fury, Matthew; Ganly, Ian; Wang, Tony J. C.; Shi, Weji; Wolden, Suzanne L.; Jackson, Andrew; Wong, Richard J.; Zhang, Zhigang; Rao, Shyam D.; Lee, Nancy Y.; 2014	A nomogram to predict loco-regional control after re-irradiation for head and neck cancer	ORN without information about localisation
Rusthoven, Kyle E.; Feigenberg, Steven J.; Raben, David; Kane, Madeleine; Song, John I.; Nicolaou, Nicos; Mehra, Ranee; Burtness, Barbara; Ridge, John; Swing, Robyn; Lango, Miriam; Cohen, Roger; Jimeno, Antonio; Chen, Changhu; 2010	Initial results of a Phase I dose-escalation trial of concurrent and maintenance erlotinib and reirradiation for recurrent and new primary head-and-neck cancer	ORN without information about localisation
Scolari, Chiara; Buchali, André; Franzen, Achim; Förster, Robert; Windisch, Paul; Bodis, Stephan; Zwahlen, Daniel R.; Schröder, Christina; 2023	Re-irradiation for head and neck cancer: outcome and toxicity analysis using a prospective single institution database	ORN without information about localisation
Sher, David J.; Haddad, Robert I.; Norris, Charles M. [JR]; Posner, Marshall R.; Wirth, Lori J.; Goguen, Laura A.; Annino, Donald; Balboni, Tracy; Allen, Aaron; Tishler, Roy B.; 2010	Efficacy and toxicity of reirradiation using intensity-modulated radiotherapy for recurrent or second primary head and neck cancer	ORN without information about localisation

Stanisce, L.; Koshkareva, Y.; Xu, Q. Y.; Patel, A.; Squillante, C.; Ahmad, N.; Rajagopalan, K.; Kubicek, G. J.; 2018	Stereotactic Body Radiotherapy Treatment for Recurrent, Previously Irradiated Head and Neck Cancer	ORN without information about localisation
Strnad, V.; Lotter, M.; Kreppner, S.; Fietkau, R.; 2015	Reirradiation for recurrent head and neck cancer with salvage interstitial pulsed-dose-rate brachytherapy	ORN without information about localisation
Tao, Yungan; Faivre, Laura; Laprie, Anne; Boisselier, Pierre; Ferron, Christophe; Jung, Guy Michel; Racadot, Séverine; Gery, Bernard; Even, Caroline; Breuskin, Ingrid; Bourhis, Jean; Janot, François; 2018	Randomized trial comparing two methods of re-irradiation after salvage surgery in head and neck squamous cell carcinoma: Once daily split-course radiotherapy with concomitant chemotherapy or twice daily radiotherapy with cetuximab	ORN without information about localisation
Thomas, J.; Wang, H.; Da Clump; Ferris, R. L.; Duvvuri, U.; Ohr, J.; Heron, de; 2020	Long-Term Patient-Reported Quality of Life After Stereotactic Body Radiation Therapy for Recurrent, Previously-Irradiated Head and Neck Cancer	ORN without information about localisation
Tian, Yun-Ming; Guan, Ying; Xiao, Wei-Wei; Zeng, Lei; Liu, Shuai; Lu, Tai-Xiang; Zhao, Chong; Han, Fei; 2016	Long-term survival and late complications in intensity-modulated radiotherapy of locally recurrent T1 to T2 nasopharyngeal carcinoma	ORN without information about localisation
Tortochaux, Jacques; Tao, Yungan; Tournay, Elodie; Lapeyre, Michel; Lesaunier, Francois; Bardet, Etienne; Janot, François; Lusinchi, Antoine; Benhamou, Ellen; Bontemps, Patrick; Maingon, Philippe; Calais, Gilles; Daly-Schveitzer, Nicolas; Verrelle, Pierre; Bourhis, Jean; 2011	Randomized phase III trial (GORTEC 98-03) comparing re-irradiation plus chemotherapy versus methotrexate in patients with recurrent or a second primary head and neck squamous cell carcinoma, treated with a palliative intent	ORN without information about localisation
Vargo, John A.; Heron, Dwight E.; Ferris, Robert L.; Rwigema, Jean-Claude M.; Kalash, Ronny; Wegner, Rodeny E.; Ohr, James; Burton, Steven; 2014	Examining tumor control and toxicity after stereotactic body radiotherapy in locally recurrent previously irradiated head and neck cancers: implications of treatment duration and tumor volume	ORN without information about localisation
Vargo, John A.; Kubicek, Gregory J.; Ferris, Robert L.; Duvvuri, Umamaheswar; Johnson, Jonas T.; Ohr, James; Clump, David A.; Burton, Steven; Heron, Dwight E.; 2014	Adjuvant stereotactic body radiotherapy±cetuximab following salvage surgery in previously irradiated head and neck cancer	ORN without information about localisation
Vargo, John A.; Ward, Matthew C.; Caudell, Jimmy J.; Riaz, Nadeem; Dunlap, Neal E.; Isrow, Derek; Zakem, Sara J.; Dault, Joshua; Awan, Musaddiq J.; Higgins, Kristin A.; Hassanadeh, Comron; Beitler, Jonathan J.; Reddy, Chandana A.; Marcrom, Samuel; Boggs, Drexell H.; Bonner, James A.; Yao, Min; Machtay, Mitchell; Siddiqui, Farzan; Trotti, Andy M.; Lee, Nancy Y.; Koyfman, Shlomo A.; Ferris, Robert L.; Heron, Dwight E.; 2018	A Multi-institutional Comparison of SBRT and IMRT for Definitive Reirradiation of Recurrent or Second Primary Head and Neck Cancer	ORN without information about localisation
Velez, Maria A.; Veruttipong, Darlene; Wang, Pin-Chieh; Chin, Robert; Beron, Philip; Abemayor, Elliot; St John, Maie; Chen, Allen M.; 2017	Re-irradiation for recurrent and second primary cancers of the head and neck	ORN without information about localisation
Wald, P.; Grecula, J.; Walston, S.; Wei, L.; Bhatt, A.; Martin, D.; Bonomi, M.; Rocco, J.; Old, M.; Teknos, T.; Blakaj, D.; 2019	Intraoperative electron beam radiotherapy for locoregionally persistent or recurrent head and neck cancer	ORN without information about localisation
Ward, Matthew C.; Riaz, Nadeem; Caudell, Jimmy J.; Dunlap, Neal E.; Isrow, Derek; Zakem, Sara J.; Dault, Joshua; Awan, Musaddiq J.; Vargo, John A.; Heron, Dwight E.; Higgins, Kristin A.; Beitler, Jonathan J.; Marcrom, Samuel; Boggs, Drexell H.; Hassanzadeh, Comron; Reddy, Chandana A.; Bonner, James A.; Yao, Min; Machtay, Mitchell; Siddiqui, Farzan; Trotti, Andy M.; Lee, Nancy Y.; Koyfman, Shlomo A.; 2018	Refining Patient Selection for Reirradiation of Head and Neck Squamous Carcinoma in the IMRT Era: A Multi-institution Cohort Study by the MIRI Collaborative	ORN without information about localisation
Watkins, John M.; Shirai, Keisuke S.; Wahlquist, Amy E.; Stuart, Robert K.; Chaudhary, Uzair B.; Garrett-Mayer, Elizabeth; Day, Terry A.; Gillespie, M. Boyd; Sharma, Anand K.; 2009	Toxicity and survival outcomes of hyperfractionated split-course reirradiation and daily concurrent chemotherapy in locoregionally recurrent, previously irradiated head and neck cancers	ORN without information about localisation
Yamazaki, Hideya; Demizu, Yusuke; Okimoto, Tomoaki; Ogita, Mikio; Himei, Kengo; Nakamura, Satoaki; Suzuki, Gen; Yoshida, Ken; Kotsuma, Tadayuki; Yoshioka, Yasuo; Oh, Ryoongjin; 2017	Reirradiation for recurrent head and neck cancers using charged particle or photon radiotherapy	ORN without information about localisation

Agas, Ryan Anthony F.; Yu, Kelvin Ken L.; Sogono, Paolo G.; Co, Lester Bryan A.; Jacinto, J C Kennetth M; Bacorro, Warren R.; Mejia, Michael Benedict A.; 2019	Reirradiation for Recurrent Nasopharyngeal Carcinomas: Experience From an Academic Tertiary Center in a Low- to Middle-Income Country	No information about ORN
Anand, Anil Kumar; Dua, Bharat; Bansal, Anil Kumar; Singh, Heigrujam Malhotra; Verma, Amit; Kumar, Amit; 2020	Stereotactic ablative radiotherapy (SABR) for recurrent and previously irradiated head and neck cancers	No information about ORN
Ayukawa, Fumio; Shibuya, Hitoshi; Yoshimura, Ryo-ichi; Watanabe, Hiroshi; Miura, Masahiko; 2007	Curative brachytherapy for recurrent/residual tongue cancer	No information about ORN
Azami, Yusuke; Hayashi, Yuichiro; Nakamura, Tatsuya; Kimura, Kanako; Yamaguchi, Hisashi; Ono, Takashi; Takayama, Kanako; Hirose, Katsumi; Yabuuchi, Tomonori; Suzuki, Motohisa; Hatayama, Yoshiomi; Hareyama, Masato; Kikuchi, Yasuhiro; Fuwa, Nobukazu; 2019	Proton Beam Therapy for Locally Recurrent Parotid Gland Cancer	No information about ORN
Biagioli, Matthew C.; Harvey, Mark; Roman, Eloy; Raez, Luis E.; Wolfson, Aaron H.; Mutyala, Subhakar; Han, Hyo S.; Markoe, Arnold; 2007	Intensity-modulated radiotherapy with concurrent chemotherapy for previously irradiated, recurrent head and neck cancer	No information about ORN
Bonomo, Pierluigi; Cipressi, Samantha; Iermano, Carmine; Bonucci, Ivano; Masi, Laura; Doro, Raffaela; Favuzza, Virginia; Paiar, Fabiola; Simontacchi, Gabriele; Meattini, Icro; Greto, Daniela; Agresti, Benedetta; Livi, Lorenzo; Biti, Giampaolo; 2014	Salvage stereotactic re-irradiation with CyberKnife for locally recurrent head and neck cancer: a single center experience	No information about ORN
Bottke, Dirk; Bathe, Kathrin; Wiegel, Thomas; Hinkelbein, Wolfgang; 2007	Phase I trial of radiochemotherapy with bendamustine in patients with recurrent squamous cell carcinoma of the head and neck	No information about ORN
Chan, Oscar S. H.; Sze, Henry C. K.; Lee, Michael C. H.; Chan, Lucy L. K.; Chang, Amy T. Y.; Lee, Sarah W. M.; Hung, Wai Man; Lee, Anne W. M.; Ng, Wai Tong; 2017	Reirradiation with intensity-modulated radiotherapy for locally recurrent T3 to T4 nasopharyngeal carcinoma	No information about ORN
Cheah, Soon Keat; Lau, Fen Nee; Yusof, Mastura Md; Phua, Vincent Chee Ee; 2014	Treatment outcome with brachytherapy for recurrent nasopharyngeal carcinoma	No information about ORN
Chen, Allen M.; Cao, Minsong; Hsu, Sophia; Lamb, James; Mikaeilian, Argin; Yang, Yingli; Agazaryan, Nzhde; Low, Daniel A.; Steinberg, Michael L.; 2017	Magnetic resonance imaging guided reirradiation of recurrent and second primary head and neck cancer	No information about ORN
Chen, Hai-yan; Ma, Xiu-mei; Ye, Ming; Hou, Yan-li; Xie, Hua-Ying; Bai, Yong-rui; 2013	Effectiveness and toxicities of intensity-modulated radiotherapy for patients with locally recurrent nasopharyngeal carcinoma	No information about ORN
Chen, Yi-Jen; Kuo, Jeffrey V.; Ramsinghani, Nilam S.; Al-Ghazi, Muthana S. A. L.; 2002	Intensity-modulated radiotherapy for previously irradiated, recurrent head-and-neck cancer	No information about ORN
Choi, Seo Hee; Chang, Jee Suk; Choi, Jinhyun; Park, So Hyun; Keum, Ki Chang; Park, Kyung Ran; Lee, Chang Geol; 2018	Re-irradiation Using Intensity-modulated Radiotherapy for Recurrent and Second Primary Head and Neck Cancer	No information about ORN
Chua, Daniel T. T.; Wu, Shao-Xiong; Lee, Victor; Tsang, Janice; 2009	Comparison of single versus fractionated dose of stereotactic radiotherapy for salvaging local failures of nasopharyngeal carcinoma: a matched-cohort analysis	No information about ORN
Cohen, Ezra E. W.; Rosine, Dominique; Haraf, Daniel J.; Loh, Elwyn; Shen, Liji; Lusinchi, Antoine; Vokes, Everett E.; Bourhis, Jean; 2007	Phase I trial of tirapazamine, cisplatin, and concurrent accelerated boost reirradiation in patients with recurrent head and neck cancer	No information about ORN
Comet, B.; Kramar, A.; Faivre-Pierret, M.; Dewas, S.; Coche-Dequeant, B.; Degardin, M.; Lefebvre, J. L.; Lacornerie, T.; Lartigau, E. F.; 2012	Salvage Stereotactic Reirradiation With or Without Cetuximab for Locally Recurrent Head-and-Neck Cancer: A Feasibility Study	No information about ORN
Dizman, Aysen; Coskun-Breuneval, Mehtap; Altinisik-Inan, Gonca; Olcay, Gokce Kaan; Cetindag, Mehmet Faik; Guney, Yildiz; 2014	Reirradiation with robotic stereotactic body radiotherapy for recurrent nasopharyngeal carcinoma	No information about ORN
Echevarria, Michelle I.; Yang, George Q.; Chen, Dung-Tsa; Kirtane, Kedar; Russell, Jeffery; Kish, Julie; Muzaffar, Jameel; Otto, Kristen; Padhya, Tapan; McMullen, Caitlin; Patel, Krupal; Chung, Christine H.; Caudell, Jimmy J.; 2023	Phase 1 Dose Escalation of Stereotactic Body Radiation Therapy and Concurrent Cisplatin for Reirradiation of Unresectable, Recurrent Squamous Cell Carcinoma of the Head and Neck	No information about ORN

Embring, A.; Onjukka, E.; Mercke, C.; Lax, I.; Berglund, A.; Bornedal, S.; Wennberg, B.; Friesland, S.; 2020	Overlapping volumes in re-irradiation for head and neck cancer - an important factor for patient selection	No information about ORN
Fan, D.; Kang, J. J.; Fan, M.; Wang, H. L.; Lee, A.; Yu, Y.; Chen, L. D.; Tsai, C. J.; McBride, S. M.; Riaz, N.; Gelblum, D. Y.; Neal, B. P.; Fetten, J.; La Dunn; Michel, L. S.; Boyle, J. O.; Cohen, M. A.; Roman, B. R.; Ganly, I.; Singh, B.; Wong, R. J.; Sherman, E. J.; Lee, N. Y.; 2020	Last-line local treatment with the Quad Shot regimen for previously irradiated head and neck cancers	No information about ORN
Garg, Shivank; Kilburn, Jeremy M.; Lucas, John T. [JR]; Randolph, David; Urbanic, James J.; Hinson, William H.; Kearns, William T.; Porosnicu, Mercedes; Greven, Kathryn; 2016	Reirradiation for second primary or recurrent cancers of the head and neck: Dosimetric and outcome analysis	No information about ORN
Goldstein, D. P.; Karnell, L. H.; Yao, M.; Chamberlin, G. P.; Nguyen, T. X.; Funk, G. F.; 2008	Outcomes following reirradiation of patients with head and neck cancer	No information about ORN
Gordon, K.; Gulidov, I.; Semenov, A.; Golovanova, O.; Koryakin, S.; Makeenkova, T.; Ivanov, S.; Kaprin, A.; 2021	Proton re-irradiation of unresectable recurrent head and neck cancers	No information about ORN
Grimard, L.; Esche, B.; Lamothe, A.; Cygler, J.; Spaans, J.; 2006	Interstitial low-dose-rate brachytherapy in the treatment of recurrent head and neck malignancies	No information about ORN
Guan, Ying; Liu, Shuai; Wang, Han-Yu; Guo, Ying; Xiao, Wei-Wei; Chen, Chun-Yan; Zhao, Chong; Lu, Tai-Xiang; Han, Fei; 2016	Long-term outcomes of a phase II randomized controlled trial comparing intensity-modulated radiotherapy with or without weekly cisplatin for the treatment of locally recurrent nasopharyngeal carcinoma	No information about ORN
Haraf, D. J.; Weichselbaum, R. R.; Vokes, E. E.; 1996	Re-irradiation with concomitant chemotherapy of unresectable recurrent head and neck cancer: a potentially curable disease	No information about ORN
Heron, de; Rwigema, J. C.M.; Gibson, M. K.; Burton, S. A.; Quinn, A. E.; Ferris, R. L.; 2011	Concurrent Cetuximab With Stereotactic Body Radiotherapy for Recurrent Squamous Cell Carcinoma of the Head and Neck *A Single Institution Matched Case-Control Study*	No information about ORN
Hu, Jiyi; Huang, Qingting; Gao, Jing; Guan, Xiyin; Hu, Weixu; Yang, Jing; Qiu, Xianxin; Chen, Mingyuan; Kong, Lin; Lu, Jiade J.; 2020	Clinical outcomes of carbon-ion radiotherapy for patients with locoregionally recurrent nasopharyngeal carcinoma	No information about ORN
Hua, Yi-Jun; Han, Fei; Lu, Li-Xia; Mai, Hai-Qiang; Guo, Xiang; Hong, Ming-Huang; Lu, Tai-Xiang; Zhao, Chong; 2012	Long-term treatment outcome of recurrent nasopharyngeal carcinoma treated with salvage intensity modulated radiotherapy	No information about ORN
Huang, Rui; Li, Zhihui; Yang, Fan; Zhang, Yu; Jiang, Yanhui; Li, Churong; Gao, Hui; Li, Guanghui; Liu, Yimin; Guo, Wenyan; Yang, Dingqiang; Zhang, Shichuan; Li, Jie; Wen, Hao; Lang, Jinyi; Zhang, Peng; 2023	Efficacy and safety of PLDR-IMRT for the re-irradiation of recurrent NPC: A prospective, single-arm, multicenter trial	No information about ORN
Huang, T. L.; Chuang, H. C.; Tsai, M. H.; Chien, C. Y.; Su, Y. Y.; Lin, Y. T.; Yang, C. H.; Lai, C. C.; Li, S. H.; Fang, F. M.; 2022	Stereotactic body radiotherapy plus cetuximab for previously irradiated un-resectable head and neck cancer	No information about ORN
Iseli, Tim A.; Iseli, Claire E.; Rosenthal, Eben L.; Caudell, Jimmy J.; Spencer, Sharon A.; Magnuson, J. Scott; Smith, Angelia N.; Carroll, William R.; 2009	Postoperative reirradiation for mucosal head and neck squamous cell carcinomas	No information about ORN
Ji, Z.; Jiang, Y. L.; Tian, S. Q.; Guo, F. X.; Peng, R.; Xu, F.; Sun, H. T.; Fan, J. H.; Wang, J. J.; 2019	The Effectiveness and Prognostic Factors of CT-Guided Radioactive I-125 Seed Implantation for the Treatment of Recurrent Head and Neck Cancer After External Beam Radiation Therapy	No information about ORN
Kasperts, Nicolien; Slotman, Ben J.; Leemans, C. Rene; Bree, Remco de; Doornaert, Patricia; Langendijk, Johannes A.; 2006	Results of postoperative reirradiation for recurrent or second primary head and neck carcinoma	No information about ORN
Kharouta, Michael; Zender, Chad; Podder, Tarun; Rezaee, Rod; Lavertu, Pierre; Fowler, Nicole; Thuener, Jason; Li, Shawn; Clancy, Kate; Xu, Zhengzheng; Yao, Min; 2021	Permanent Interstitial Cesium-131 Brachytherapy in Treating High-Risk Recurrent Head and Neck Cancer: A Prospective Pilot Study	No information about ORN

Kong, Fangfang; Zhou, Junjun; Du, Chengrun; He, Xiayun; Kong, Lin; Hu, Chaosu; Ying, Hongmei; 2018	Long-term survival and late complications of intensity-modulated radiotherapy for recurrent nasopharyngeal carcinoma	No information about ORN
Kong, Lin; Wang, Lei; Shen, Chunying; Hu, Chaosu; Lu, Jiade J.; 2016	Salvage Intensity-Modulated Radiation Therapy (IMRT) for Locally Recurrent Nasopharyngeal Cancer after Definitive IMRT: A Novel Scenario of the Modern Era	No information about ORN
Koukourakis, Ioannis M.; Giakzidis, Axiotis G.; Koukourakis, Michael I.; 2023	Anti-PD-1 immunotherapy with dose-adjusted ultra-hypofractionated re-irradiation in patients with locoregionally recurrent head and neck cancer	No information about ORN
Koutcher, Lawrence; Lee, Nancy; Zelefsky, Michael; Chan, Kelvin; Cohen, Gilad; Pfister, David; Kraus, Dennis; Wolden, Suzanne; 2010	Reirradiation of locally recurrent nasopharynx cancer with external beam radiotherapy with or without brachytherapy	No information about ORN
Kress, M. A.S.; Sen, N.; Unger, K. R.; Lominska, C. E.; Deeken, J. F.; Davidson, B. J.; Newkirk, K. A.; Hwang, J.; Harter, K. W.; 2015	Safety and efficacy of hypofractionated stereotactic body reirradiation in head and neck cancer: Long-term follow-up of a large series	No information about ORN
Kwong, D. L.; Wei, W. I.; Cheng, A. C.; Choy, D. T.; Lo, A. T.; Wu, P. M.; Sham, J. S.; 2001	Long term results of radioactive gold grain implantation for the treatment of persistent and recurrent nasopharyngeal carcinoma	No information about ORN
Langendijk, Johannes A.; Kasperts, Nicolien; Leemans, Charles R.; Doornaert, Patricia; Slotman, Ben J.; 2006	A phase II study of primary reirradiation in squamous cell carcinoma of head and neck	No information about ORN
Langer, Corey J.; Harris, Jonathan; Horwitz, Eric M.; Nicolaou, Nicos; Kies, Merrill; Curran, Walter; Wong, Stuart; Ang, Kian; 2007	Phase II study of low-dose paclitaxel and cisplatin in combination with split-course concomitant twice-daily reirradiation in recurrent squamous cell carcinoma of the head and neck: results of Radiation Therapy Oncology Group Protocol 9911	No information about ORN
Lartigau, E. F.; Tresch, E.; Thariat, J.; Graff, P.; Coche-Dequeant, B.; Benezery, K.; Schiappacasse, L.; Degardin, M.; Bondiau, P. Y.; Peiffert, D.; Lefebvre, J. L.; Lacornerie, T.; Kramar, A.; 2013	Multi institutional phase II study of concomitant stereotactic reirradiation and cetuximab for recurrent head and neck cancer	No information about ORN
Law, Stephen C. K.; Lam, Wing-Kit; Ng, Man-Fai; Au, Siu-Kie; Mak, Wing-Tai; Lau, Wai-Hon; 2002	Reirradiation of nasopharyngeal carcinoma with intracavitary mold brachytherapy: an effective means of local salvage	No information about ORN
Lee, A. W.; Foo, W.; Law, S. C.; Poon, Y. F.; Sze, W. M.; O, S. K.; Tung, S. Y.; Lau, W. H.; 1997	Reirradiation for recurrent nasopharyngeal carcinoma: factors affecting the therapeutic ratio and ways for improvement	No information about ORN
Lee, Nancy; Chan, Kelvin; Bekelman, Justin E.; Zhung, Joanne; Mechalakos, James; Narayana, Ashwatha; Wolden, Suzanne; Venkatraman, Ennapadam S.; Pfister, David; Kraus, Dennis; Shah, Jatin; Zelefsky, Michael J.; 2007	Salvage re-irradiation for recurrent head and neck cancer	No information about ORN
Lengyel, Erzsébet; Baricza, Károly; Somogyi, Andreás; Olajos, Judit; Pápai, Zsuzsanna; Godény, Mária; Németh, Gyorgy; Esik, Olga; 2003	Reirradiation of locally recurrent nasopharyngeal carcinoma	No information about ORN
Leung, T. W.; Tung, S. Y.; Sze, W. K.; Sze, W. M.; Wong, V. Y.; O, S. K.; 2000	Salvage brachytherapy for patients with locally persistent nasopharyngeal carcinoma	No information about ORN
Li, J. C.; Hu, C. S.; Jiang, G. L.; Mayr, N. A.; Wang, J. Z.; He, X. Y.; Wu, Y. Ru; 2006	Dose escalation of three-dimensional conformal radiotherapy for locally recurrent nasopharyngeal carcinoma: a prospective randomised study	No information about ORN
Li, Yue; Jiang, Yuliang; Wang, Junjie; 2023	Safety and efficacy of CT-guided radioactive iodine-125 seed implantation as a salvage treatment for recurrent head and neck cancer after two or more courses of radiotherapy	No information about ORN
Lin, Cheng; Lu, Qiongjiao; Chen, Yuebing; Chen, Xiaoqiang; Huang, Yiping; Zhong, Hangyan; Peng, Xiane; Hu, Cairong; Chen, Bijuan; Lin, Shaojun; Zong, Jingfeng; 2024	Failure patterns and individualized treatment plans of reirradiation for inoperable locally recurrent nasopharyngeal carcinoma	No information about ORN
Lin, R.; Slater, J. D.; Yonemoto, L. T.; Grove, R. I.; Teichman, S. L.; Watt, D. K.; Slater, J. M.; 1999	Nasopharyngeal carcinoma: repeat treatment with conformal proton therapy--dose-volume histogram analysis	No information about ORN
Lin, Yanzhu; Ouyang, Yi; Lu, Zhiyuan; Liu, Yonghong; Chen, Kai; Cao, Xinping; 2019	Long-Term Outcomes of Three-Dimensional High-Dose-Rate	No information about ORN

	Brachytherapy for Locally Recurrent Early T-Stage Nasopharyngeal Carcinoma	
Liu, Feng; Xiao, Jian-Ping; Xu, Ying-Jie; Zhang, Ye; Xu, Guo-Zhen; Gao, Li; Yi, Jun-Lin; Luo, Jing-Wei; Huang, Xiao-Dong; Huan, Fu-Kui; Fang, Hao; Wan, Bao; Li, Ye-Xiong; 2012	Fractionated stereotactic radiotherapy with vagina carotica protection technique for local residual nasopharyngeal carcinoma after primary radiotherapy	No information about ORN
Liu, T. X.; Sun, Q. Q.; Hua, Y. H.; Tao, C. J.; Jiang, F.; 2024	PD-1 Inhibitors Combined with Chemotherapy versus Re-irradiation/chemoradiotherapy for Unresectable Locally Recurrent T3-4 Nasopharyngeal Carcinoma: A Retrospective Study	No information about ORN
Lu, Tai-Xiang; Mai, Wei-Yuan; Teh, Bin S.; Zhao, Chong; Han, Fei; Huang, Yin; Deng, Xiao-Wu; Lu, Li-Xia; Huang, Shao-Min; Zeng, Zhi-Fan; Lin, Cheng-Guang; Lu, Hsin H.; Chiu, J. Kam; Carpenter, L. Steven; Grant, Walter H.; Woo, Shiao Y.; Cui, Nan-Ji; Butler, E. Brian; 2004	Initial experience using intensity-modulated radiotherapy for recurrent nasopharyngeal carcinoma	No information about ORN
Machtay, M.; Di Rosenthal; Chalian, A. A.; Lustig, R.; Hershock, D.; Miller, L.; Weinstein, G. S.; Weber, R. S.; 2004	Pilot study of postoperative reirradiation, chemotherapy, and amifostine after surgical salvage for recurrent head-and-neck cancer	No information about ORN
Mallick, S.; Gandhi, A. K.; Joshi, N. P.; Pandit, S.; Bhasker, S.; Sharma, A.; Thakar, A.; Mohanti, B. K.; 2014	Re-irradiation in head and neck cancers: an Indian tertiary cancer centre experience	No information about ORN
Margalit, Danielle N.; Schoenfeld, Jonathan D.; Rawal, Bhupendra; Haddad, Robert I.; Catalano, Paul J.; Goguen, Laura A.; Chau, Nicole G.; Rabinowits, Guilherme; Lorch, Jochen H.; Annino, Donald J.; Tishler, Roy B.; 2017	Patient-oriented toxicity endpoints after head and neck reirradiation with intensity modulated radiation therapy	No information about ORN
McDonald, M. W.; Zolali-Meybodi, O.; Lehnert, S. J.; Estabrook, N. C.; Liu, Y.; Cohen-Gadol, A. A.; Moore, M. G.; 2016	Reirradiation of Recurrent and Second Primary Head and Neck Cancer With Proton Therapy	No information about ORN
Mitsuhashi, N.; Sakurai, H.; Takahashi, M.; Maebayashi, K.; Tamaki, Y.; Hashida, I.; Akimoto, T.; Nakamoto, S.; Takahashi, T.; Hayakawa, K.; 1995	Prognostic factors for loco-regional control and outcome of re-irradiation for patients with poorly-differentiated squamous cell carcinoma of the nasopharynx	No information about ORN
Nag, S.; Schuller, D. E.; Martinez-Monge, R.; Rodriguez-Villalba, S.; Grecula, J.; Bauer, C.; 1998	Intraoperative electron beam radiotherapy for previously irradiated advanced head and neck malignancies	No information about ORN
Nagar, Yoodhvir Singh; Singh, Shalini; Datta, Niloy Ranjan; 2004	Chemo-reirradiation in persistent/recurrent head and neck cancers	No information about ORN
Nakamura, Tatsuya; Kodaira, Takeshi; Tachibana, Hiroyuki; Tomita, Natsuo; Tomoda, Takuya; Nakahara, Rie; Inokuchi, Haruo; Mizoguchi, Nobutaka; Takada, Akinori; Fuwa, Nobukazu; 2008	Chemoradiotherapy for locally recurrent nasopharyngeal carcinoma: treatment outcome and prognostic factors	No information about ORN
Nishioka, T.; Shirato, H.; Kagei, K.; Fukuda, S.; Hashimoto, S.; Ohmori, K.; 2000	Three-dimensional small-volume irradiation for residual or recurrent nasopharyngeal carcinoma	No information about ORN
Öksüz, Didem Çolpan; Meral, Gülşen; Uzel, Ömer; Çağatay, Pembe; Turkan, Sedat; 2004	Reirradiation for locally recurrent nasopharyngeal carcinoma: Treatment results and prognostic factors	No information about ORN
Pai, Ping-Ching; Chuang, Chi-Cheng; Wei, Kuo-Chen; Tsang, Ngan-Ming; Tseng, Chen-Kan; Chang, Chen-Nen; 2002	Stereotactic radiosurgery for locally recurrent nasopharyngeal carcinoma	No information about ORN
Pan, Rongqiang; Wang, Jingming; Qi, Feng; Liu, Ruizhen; 2017	Dosimetric comparison and observation of three-dimensional conformal radiotherapy for recurrent nasopharyngeal carcinoma	No information about ORN
Peponi, E.; Balta, S.; Tasiou, I.; Gogou, P.; Capizzello, A.; Pitouli, E.; Tsekeris, P.; 2012	Reirradiation for recurrent head and neck carcinoma	No information about ORN
Perry, D. J.; Chan, K.; Wolden, S.; Zelefsky, M. J.; Chiu, J.; Cohen, G.; Zaider, M.; Kraus, D.; Shah, J.; Lee, N.; 2010	HIGH-DOSE-RATE INTRAOPERATIVE RADIATION THERAPY FOR RECURRENT HEAD-AND-NECK CANCER	No information about ORN
Phuong, C.; Pham, A.; Batth, S. S.; Tsao-Wei, D.; Schechter, N.; Jennelle, R. L.; Trakul, N.; Garsa, A.; 2019	Challenges in the re-irradiation of locally advanced head and neck cancers: outcomes and toxicities	No information about ORN

Pollard, Courtney 3rd; Nguyen, Theresa P.; Ng, Sweet Ping; Frank, Steven J.; Garden, Adam S.; Gunn, Gary B.; Fuller, Clifton D.; Beadle, Beth M.; Morrison, William H.; Shah, Shah J.; Wang, He; Tung, Sam; Wang, Conjun; Ginsberg, Lawrence D.; Zafereo, Mark E.; Sturgis, Erich M.; Su, Shirley Y.; Hanna, Ehab Y.; Rosenthal, David I.; Phan, Jack; 2017	Clinical outcomes after local field conformal reirradiation of patients with retropharyngeal nodal metastasis	No information about ORN
Poon, Donald; Yap, Swee-Peng; Wong, Zee-Wan; Cheung, Yin-Bun; Leong, Swan-Swan; Wee, Joseph; Tan, Terence; Fong, Kam-Weng; Chua, Eu-Tiong; Tan, Eng-Huat; 2004	Concurrent chemoradiotherapy in locoregionally recurrent nasopharyngeal carcinoma	No information about ORN
Puebla, F.; Lopez Guerra, J. L.; Garcia Ramirez, J. M.; Matute, R.; Marrone, I.; Miguez, C.; Sevillano, D.; Sanchez-Reyes, A.; Del Rivin Campo, E.; Praena-Fernandez, J. M.; Azinovic, I.; 2015	Effectiveness and toxicity of helical tomotherapy for patients with locally recurrent nasopharyngeal carcinoma	No information about ORN
Qiu, Sufang; Lin, Shaojun; Tham, Ivan W. K.; Pan, Jianji; Lu, Jun; Lu, Jiade J.; 2012	Intensity-modulated radiation therapy in the salvage of locally recurrent nasopharyngeal carcinoma	No information about ORN
Roeder, Falk; Zwicker, Felix; Saleh-Ebrahimi, Ladan; Timke, Carmen; Thieke, Christian; Bischof, Marc; Debus, Juergen; Huber, Peter E.; 2011	Intensity modulated or fractionated stereotactic reirradiation in patients with recurrent nasopharyngeal cancer	No information about ORN
Saba, Nabil F.; Wong, Stuart J.; Nasti, Tahseen; McCook-Veal, Ashley Alesia; McDonald, Mark W.; Stokes, William A.; Anderson, Allyson M.; Ekpenyong, Asari; Rupji, Manali; Abousaud, Marin; Rudra, Soumon; Bates, James E.; Remick, Jill S.; Joshi, Nikhil P.; Woody, Neil M.; Awan, Musaddiq; Geiger, Jessica L.; Shreenivas, Aditya; Samsa, Julia; Ward, Matthew Christopher; Schmitt, Nicole C.; Patel, Mihir R.; Higgins, Kristin A.; Teng, Yong; Steuer, Conor E.; Shin, Dong M.; Liu, Yuan; Ahmed, Rafi; Koyfman, Shlomo A.; 2024	Intensity-Modulated Reirradiation Therapy With Nivolumab in Recurrent or Second Primary Head and Neck Squamous Cell Carcinoma: A Nonrandomized Controlled Trial	No information about ORN
Schleifenbaum, J. K.; Morgenthaler, J.; Sharma, S. J.; Klussmann, J. P.; Linde, P.; Wegen, S.; Rosenbrock, J.; Baues, C.; Fokas, E.; Khor, R.; Ng, S. P.; Marnitz, S.; Trommer, M.; 2025	Optimising (re-)irradiation for locally recurrent head and neck cancer: impact of dose-escalation, salvage surgery, PEG tube and biomarkers on oncological outcomes-a single centre analysis	No information about ORN
Shikama, Naoto; Kumazaki, Yu; Tsukamoto, Nobuhiro; Ebara, Takeshi; Makino, Soichi; Abe, Takanori; Nakahira, Mitsuhiko; Sugasawa, Masashi; Kato, Shingo; 2013	Validation of nomogram-based prediction of survival probability after salvage re-irradiation of head and neck cancer	No information about ORN
Shin, Seong Soo; Ahn, Yong Chan; Lim, Do Hoon; Park, Won; Huh, Seung Jae; 2004	High dose 3-dimensional re-irradiation for locally recurrent nasopharyngeal cancer	No information about ORN
Slater, Noah N.; Farsi, Soroush; Rogers, Ashton L.; Herberger, Lindsey; Penagaricano, Jose; McKee, Steven; King, Deanne; Samanta, Santanu; Sunde, Jumin; Vural, Emre; Moreno, Mauricio A.; 2024	Reirradiation in head and neck squamous cell carcinoma; prognostic indicators, oncologic and functional outcomes	No information about ORN
Teckie, S.; Scala, L. M.; Ho, F.; Wolden, S.; Chiu, J.; Cohen, G. N.; Wong, R.; Ganly, I.; Zelefsky, M. J.; Lee, N. Y.; 2013	High-dose-rate intraoperative brachytherapy and radical surgical resection in the management of recurrent head-and-neck cancer	No information about ORN
Tian, Yun-Ming; Huang, Wei-Zeng; Yuan, Xia; Bai, Li; Zhao, Chong; Han, Fei; 2017	The challenge in treating locally recurrent T3-4 nasopharyngeal carcinoma: the survival benefit and severe late toxicities of re-irradiation with intensity-modulated radiotherapy	No information about ORN
Unger, K. R.; Lominska, C. E.; Deeken, J. F.; Davidson, B. J.; Newkirk, K. A.; Gagnon, G. J.; Hwang, J.; Slack, R. S.; Noone, am; Harter, K. W.; 2010	FRACTIONATED STEREOTACTIC RADIOSURGERY FOR REIRRADIATION OF HEAD-AND-NECK CANCER	No information about ORN
Vargo, John A.; Ferris, Robert L.; Ohr, James; Clump, David A.; Davis, Kara S.; Duvvuri, Umamaheswar; Kim, Seungwon; Johnson, Jonas T.; Bauman, Julie E.; Gibson, Michael K.; Branstetter, Barton F.; Heron, Dwight E.; 2015	A prospective phase 2 trial of reirradiation with stereotactic body radiation therapy plus cetuximab in patients with previously irradiated recurrent squamous cell carcinoma of the head and neck	No information about ORN

Vormittag, L.; Lemaire, C.; Radonjic, D.; Kornek, G.; Selzer, E.; 2012	Re-irradiation combined with capecitabine in locally recurrent squamous cell carcinoma of the head and neck. A prospective phase II trial	No information about ORN
Weng, Jingjin; Wei, Jiazhang; Si, Jinyuan; Qin, Yangda; Li, Min; Liu, Fei; Si, Yongfeng; Su, Jiping; 2017	Clinical outcomes of residual or recurrent nasopharyngeal carcinoma treated with endoscopic nasopharyngectomy plus chemoradiotherapy or with chemoradiotherapy alone: a retrospective study	No information about ORN
Wu, Shao-Xiong; Chua, Daniel T. T.; Deng, Mei-Ling; Zhao, Chong; Li, Feng-Yan; Sham, Jonathan S. T.; Wang, Han-Yu; Bao, Yong; Gao, Yuan-Hong; Zeng, Zhi-Fan; 2007	Outcome of fractionated stereotactic radiotherapy for 90 patients with locally persistent and recurrent nasopharyngeal carcinoma	No information about ORN
Yamazaki, Hideya; Suzuki, Gen; Aibe, Norihiro; Shiomi, Hiroya; Oh, Ryoong-Jin; Yoshida, Ken; Nakamura, Satoaki; Konishi, Koji; Ogita, Mikio; 2023	Reirradiation for local recurrence of oral, pharyngeal, and laryngeal cancers: a multi-institutional study	No information about ORN
Yang, Jing; Gao, Jing; Wu, Xiaodong; Hu, Jiyi; Hu, Weixu; Kong, Lin; Lu, Jiade J.; 2018	Salvage Carbon Ion Radiation Therapy for Locally Recurrent or Radiation-Induced Second Primary Sarcoma of the Head and Neck	No information about ORN
Yau, Tsz-Kok; Sze, Wai-Man; Lee, Wing-Mui; Yeung, Mei-Wan; Leung, Kim-Cheung; Hung, Wai-Man; Chan, Wing-Ip; 2004	Effectiveness of brachytherapy and fractionated stereotactic radiotherapy boost for persistent nasopharyngeal carcinoma	No information about ORN
You, Rui; Liu, You-Ping; Xie, Yu-Long; Lin, Chao; Duan, Chong-Yang; Chen, Dong-Ping; Pan, Yi; Qi, Bin; Zou, Xiong; Guo, Ling; Cao, Jing-Yu; Zhang, Yi-Nuan; Wang, Zhi-Qiang; Liu, Yong-Long; Ouyang, Yan-Feng; Wen, Kai; Yang, Qi; Xie, Ruo-Qi; Li, Hui-Feng; Duan, Xiao-Tong; Ding, Xi; Peng, Lan; Chen, Si-Yuan; Liang, Jiong-Lin; Feng, Zheng-Kai; Xia, Tian-Liang; Xie, Rui-Ling; Jiang, Rou; Gu, Chen-Mei; Liu, Rong-Zeng; Sun, Rui; Yang, Xin; Liu, Li-Zhi; Ling, Li; Liu, Qing; Ng, Wai Tong; Hua, Yi-Jun; Huang, Pei-Yu; Chen, Ming-Yuan; 2023	Hyperfractionation compared with standard fractionation in intensity-modulated radiotherapy for patients with locally advanced recurrent nasopharyngeal carcinoma: a multicentre, randomised, open-label, phase 3 trial	No information about ORN
Yu, Nathan Y.; Gamez, Mauricio E.; Hartsell, William F.; Tsai, Henry K.; Laramore, George E.; Larson, Gary L.; Simone, Charles B.; Rossi, Carl; Katz, Sanford R.; Buras, Matthew R.; Golafshar, Michael A.; Vargas, Carlos E.; Patel, Samir H.; 2019	A Multi-Institutional Experience of Proton Beam Therapy for Sinonasal Tumors	No information about ORN

Supplementary Material (H): Risk of bias assessment

		methological items for non-randomized studies								additional criteria in the case of comparative study																
MINORS		1	2	3	4	5	6	7	8	9	10	11	12													
The items are scored 0 (not reported), 1 (reported but inadequate) or 2 (reported and adequate); global ideal score for non-comparative studies: 16																	modified for ORN: JBI critical appraisal checklist for studies reporting prevalence data									
study	year	A clearly stated aim	Inclusion of consecutive patients	Prospective collection of data	Endpoints appropriate to aim of study	unbiased assessment of study endpoint	follow-up period appropriate to the aim of study	loss to follow-up less than 5%	prospective calculation of study size	an adequate control group	contemporary groups	baseline equivalence of groups	adequate statistical analyses	total score (x/16) oder (x/24)	x/16*100		Was the sample frame appropriate to address the target population?	Were study participants sampled in an appropriate way?	Was the sample size adequate?	Were the study subjects and the setting described in detail?	Was the condition measured in a standard, reliable way for all participants?	Were valid methods used for the identification of the condition?	was the follow-up duration adequate for detection?	Were all diagnosis related stages reported?	total score (x/8)	x/8*100
Al-Wassia et al.	2015	2	2	0	2	1	2	1	0	/	/	/	/	10	62.5		yes	yes	no	yes	unclear	yes	yes	yes	6/8	75
Awan et al.	2018	2	2	2	2	1	2	2	1	/	/	/	/	14	87.5		yes	yes	no	yes	unclear	yes	yes	yes	6/8	75
Bhalavat et al.	2018	2	1	0	2	0	2	1	0	/	/	/	/	8	50.0		yes	unclear	no	yes	unclear	yes	yes	unclear	4/8	50
Bahl et al.	2018	2	0	0	2	1	0	2	0	/	/	/	/	7	43.75		yes	no	no	yes	unclear	yes	unclear	yes	4/8	50
Beddok et al.	2023	2	2	0	2	1	2	1	0	/	/	/	/	10	62.5		yes	yes	no	yes	unclear	yes	yes	yes	6/8	75
Benchalal et al.	1995	2	1	2	2	1	2	2	0	/	/	/	/	11	68.75		yes	unclear	no	yes	no	unclear	yes	unclear	3/8	37.5
Berger et al.	2010	2	1	2	2	1	2	1	2	/	/	/	/	13	81.25		yes	yes	yes	yes	unclear	yes	yes	unclear	6/8	75
Bots et al.	2017	2	2	0	2	1	2	1	0	/	/	/	/	10	62.5		yes	yes	yes	yes	unclear	yes	yes	no	6/8	75
Boustani et al.	2021	2	2	0	2	1	2	2	0	/	/	/	/	11	68.75		yes	yes	no	yes	unclear	yes	yes	no	5/8	62.5
Buglione et al.	2015	2	2	0	2	1	2	1	0	/	/	/	/	10	62.5		yes	yes	yes	yes	unclear	yes	unclear	yes	6/8	75
Bussu et al.	2024	2	2	0	2	1	2	1	0	/	/	/	/	10	62.5		yes	unclear	no	yes	yes	yes	yes	no	5/8	62.5
Cengiz et al.	2011	2	1	0	2	1	2	1	0	/	/	/	/	9	56.25		yes	unclear	no	yes	unclear	yes	yes	yes	5/8	62.5
Chen et al.	2007	2	2	0	2	1	2	1	0	/	/	/	/	10	62.5		yes	yes	yes	yes	unclear	no	yes	unclear	5/8	62.5
Chen et al.	2011	2	2	1	2	1	2	1	0	/	/	/	/	11	68.75		yes	yes	no	yes	unclear	yes	yes	no	5/8	62.5
Crevoisier et al.	2001	2	1	2	2	1	2	2	0	/	/	/	/	12	75.0		yes	yes	no	yes	unclear	yes	yes	unclear	5/8	62.5
Cvek et al.	2016	2	1	0	2	1	2	2	0	/	/	/	/	10	62.5		yes	unclear	no	yes	unclear	yes	yes	unclear	4/8	50
Dionisi et al.	2019	2	2	0	2	1	2	1	0	/	/	/	/	10	62.5		yes	yes	no	yes	unclear	yes	yes	yes	6/8	75
Domoff et al.	2015	2	1	0	2	1	2	1	0	1	2	1	2	16	66.67		yes	no	yes	yes	unclear	yes	yes	no	5/8	62.5
Duprez et al.	2009	2	1	0	2	1	2	1	0	/	/	/	/	9	56.25		yes	unclear	yes	yes	unclear	yes	yes	no	5/8	62.5
Embring et al.	2021	2	2	0	2	1	2	1	0	/	/	/	/	10	62.5		yes	yes	yes	yes	unclear	yes	yes	no	6/8	75
Hall et al.	2003	2	1	2	2	1	2	2	0	/	/	/	/	12	75.0		yes	yes	no	yes	unclear	yes	yes	unclear	5/8	62.5
Hegde et al.	2018	2	1	0	2	1	2	1	0	/	/	/	/	10	62.5		yes	unclear	no	yes	yes	yes	yes	yes	6/8	75
Hepel et al.	2005	1	0	0	2	1	2	2	0	/	/	/	/	8	50.0		yes	unclear	no	yes	unclear	no	yes	unclear	3/8	37.5
Held et al.	2019	2	2	0	2	1	2	1	0	/	/	/	/	10	62.5		yes	yes	yes	yes	yes	yes	yes	yes	8/8	100
Heron et al.	2009	2	2	2	2	1	1	1	2	/	/	/	/	13	81.25		yes	yes	no	yes	yes	unclear	unclear	unclear	4/8	50
Hsieh et al.	2024	2	2	0	2	1	2	1	0	/	/	/	/	10	62.5		yes	yes	no	yes	yes	yes	yes	unclear	6/8	75
Hoell et al.	2023	2	1	0	2	1	2	1	0	/	/	/	/	9	56.25		yes	yes	no	yes	unclear	yes	yes	unclear	5/8	62.5
Hwang et al.	1998	2	1	0	2	1	2	2	0	/	/	/	/	10	62.5		yes	yes	yes	yes	unclear	yes	yes	unclear	6/8	75
Jiang et al.	2019	2	2	0	2	1	2	2	0	/	/	/	/	10	62.5		yes	yes	yes	yes	unclear	yes	yes	yes	7/8	87.5
Jensen et al.	2010	2	1	0	2	1	2	1	0	/	/	/	/	9	56.25		yes	unclear	no	yes	unclear	yes	yes	unclear	4/8	50
Kakria et al.	2014	2	1	0	2	1	2	1	0	/	/	/	/	9	56.25		yes	unclear	no	yes	unclear	yes	yes	unclear	4/8	50
Karam et al.	2016	2	2	0	2	1	2	1	0	/	/	/	/	11	68.75		yes	yes	no	yes	unclear	yes	yes	no	5/8	62.5
Kramer et al.	2005	2	2	2	2	1	2	2	1	/	/	/	/	14	87.5		yes	yes	no	yes	unclear	yes	yes	unclear	5/8	62.5
Lee et al.	2017	2	1	1	2	1	2	1	0	1	0	1	2	14	58.34		yes	unclear	no	yes	unclear	yes	yes	no	4/8	50
Lee et al.	2016	2	2	0	2	1	2	1	0	/	/	/	/	10	62.5		yes	yes	yes	yes	unclear	yes	yes	yes	7/8	87.5
Low et al.	2006	2	1	0	2	1	2	1	0	/	/	/	/	9	56.25		yes	unclear	no	yes	yes	no	yes	unclear	4/8	50
Megahed et al.	2024	2	2	0	2	1	1	2	0	/	/	/	/	10	62.5		yes	yes	no	yes	unclear	yes	unclear	yes	5/8	62.5
Martinez-Fernández et al.	2017	2	2	2	2	1	2	1	0	/	/	/	/	12	75.0		yes	yes	yes	yes	unclear	yes	yes	no	6/8	75
Orlandi et al.	2019	2	2	0	2	1	2	2	0	/	/	/	/	11	68.75		yes	yes	yes	yes	unclear	yes	yes	no	6/8	75
Ozyigit et al.	2011	2	2	0	2	1	2	2	0	1	0	1	2	15	62.5		yes	yes	no	yes	unclear	yes	yes	no	5/8	62.5
Pialat et al.	2021	2	1	0	2	1	2	1	0	/	/	/	/	9	56.25		yes	unclear	no	yes	unclear	yes	yes	unclear	4/8	50

MINORS		methological items for non-randomized studies								additional criteria in the case of comparative study					
		1	2	3	4	5	6	7	8	9	10	11	12		
The items are scored 0 (not reported), 1 (reported but inadequate) or 2 (reported and adequate); global ideal score for non-comparative studies: 16															
**study**	**year**	A clearly stated aim	Inclusion of consecutive patients	Prospective collection of data	Endpoints appropriate to aim of study	unbiased assessment of study endpoint	follow-up period appropriate to the aim of study	loss to follow-up less than 5%	prospective calculation of study size	an adequate control group	contemporary groups	baseline equivalence of groups	adequate statistical analyses	**total score (x/16) oder (x/24)**	**x/16*100**
Ritter et al.	2016	2	2	0	2	1	2	1	0	2	1	1	2	16	66.67
Roh et al.	2009	2	1	0	2	1	2	1	0	/	/	/	/	9	56.25
Romesser et al.	2016	2	2	2	2	1	2	1	0	/	/	/	/	12	75.0
Rühle et al.	2020	2	2	0	2	1	2	1	0	/	/	/	/	10	62.5
Schaefer et al.	2000	2	2	2	2	1	2	2	0	/	/	/	/	13	81.25
Salama et al.	2006	2	2	0	2	1	2	1	0	/	/	/	/	10	62.5
Seiwert et al.	2008	2	2	2	2	1	2	1	0	/	/	/	/	12	75.0
Siddiqui et al.	2009	2	1	0	2	1	2	1	0	/	/	/	/	9	56.25
Spencer et al.	2007	2	2	2	2	1	2	1	0	/	/	/	/	12	75.0
Suh et al.	2010	2	1	0	2	1	2	2	0	/	/	/	/	10	62.5
Sulman et al.	2009	2	2	0	2	1	2	1	0	/	/	/	/	10	62.5
Takiar et al.	2016	2	2	0	2	1	2	1	0	/	/	/	/	10	62.5
Teo et al.	1998	2	1	0	2	1	2	2	0	/	/	/	/	10	62.5
Tselis et al.	2011	2	2	0	2	1	2	2	0	/	/	/	/	11	68.75
Vischioni et al.	2020	2	2	2	2	1	2	2	0	/	/	/	/	12	75.0
Voynov et al.	2006	2	2	0	2	1	2	2	0	/	/	/	/	11	68.75
Waldron et al.	2019	2	2	0	2	1	2	2	0	/	/	/	/	11	68.75
Zheng et al.	2004	2	2	0	2	1	2	1	0	1	2	2	2	17	70.83
Zwicker et al.	2011	2	2	0	2	1	2	2	0	/	/	/	/	11	68.75

modified for ORN: JBI critical appraisal checklist for studies reporting prevalence data

study	year	Was the sample frame appropriate to address the target population?	Were study participants sampled in an appropriate way?	Was the sample size adequate?	Were the study subjects and the setting described in detail?	Was the condition measured in a standard, reliable way for all participants?	Were valid methods used for the identification of the condition?	was the follow-up duration adequate for detection?	Were all diagnosis related stages reported?	**total score (x/8)**	**x/8*100**
Ritter et al.	2016	yes	yes	no	yes	unclear	yes	yes	unclear	5/8	62.5
Roh et al.	2009	yes	unclear	no	yes	yes	yes	yes	unclear	5/8	62.5
Romesser et al.	2016	yes	yes	yes	yes	yes	yes	yes	yes	8/8	100
Rühle et al.	2020	yes	yes	no	yes	unclear	yes	yes	yes	6/8	75
Schaefer et al.	2000	yes	yes	no	yes	unclear	yes	yes	unclear	5/8	62.5
Salama et al.	2006	yes	yes	yes	yes	yes	yes	yes	no	7/8	87.5
Seiwert et al.	2008	yes	yes	no	yes	unclear	yes	yes	no	5/8	62.5
Siddiqui et al.	2009	yes	unclear	no	yes	unclear	yes	yes	no	4/8	50
Spencer et al.	2007	yes	yes	yes	yes	yes	yes	yes	no	7/8	87.5
Suh et al.	2010	yes	yes	no	yes	unclear	yes	yes	yes	6/8	75
Sulman et al.	2009	yes	yes	yes	yes	unclear	unclear	yes	no	5/8	62.5
Takiar et al.	2016	yes	yes	yes	yes	unclear	yes	yes	no	6/8	75
Teo et al.	1998	yes	unclear	yes	yes	unclear	no	yes	unclear	4/8	50
Tselis et al.	2011	yes	yes	yes	yes	unclear	yes	yes	no	6/8	75
Vischioni et al.	2020	yes	yes	yes	yes	unclear	yes	yes	yes	7/8	87.5
Voynov et al.	2006	yes	yes	no	yes	unclear	yes	yes	unclear	5/8	62.5
Waldron et al.	2019	yes	yes	no	yes	unclear	yes	yes	unclear	5/8	62.5
Zheng et al.	2004	yes	yes	yes	yes	unclear	yes	yes	no	6/8	75
Zwicker et al.	2011	yes	yes	no	yes	unclear	yes	yes	yes	6/8	75
Rudzianskas et al.	2014	yes	yes	yes	yes	unclear	yes	yes	unclear	6/8	75

RoB2		Bias arising from the randomisation process	Bias due to deviations from intended interventions	Bias due to missing outcome data	Bias in measurement of the outcome	Bias in selection of the reported result	Overall bias
Rudzianskas et al.	2014	some concerns	some concerns	low	high	some concerns	some concerns

RoB2	for Rudzianskas et al. 2014	Y	PY	N	PN	NI	NA
	**1. Bias arising from the randomisation process**						
	1.1 Was the allocation sequence random?	x					
	1.2 Was the allocation sequence concealed until participants were enrolled and assigned to interventions?					x	
	1.3 Did baseline differences between intervention groups suggest a problem with the randomisation process?			x			
	Risk-of-bias judgment (low/high/**some concerns**)						
	**2. Bias due to deviations from intended interventions**						
	2.1 Were participants aware of their assigned intervention during the trial?	x					
	2.2 Were carers and people delivering the interventions aware of participants' assigned intervention during the trial?	x					
	2.3 If Y/PY/NI to 2.1 or 2.2: Were there deviations from the intended intervention that arose because of the trial context?			x			
	2.4 If Y/PY/NI to 2.3: Were these deviations likely to have affected the outcome?			x			
	2.5 If Y/PY to 2.4: Were these deviations from intended intervention balanced between groups?					x	
	2.6 Was an appropriate analysis used to estimate the effect of assignment to intervention?					x	
	2.7 If N/PN/NI to 2.6: Was there potential for a substantial impact (on the result) of the failure to analyse participants in the group to which they were randomised?		x				
	Risk-of-bias judgment (low/high/**some concerns**)						
	**3. Bias due to missing outcome data**						
	3.1 Were data for this outcome available for all, or nearly all, participants randomised?		x				
	3.2 If N/PN/NI to 3.1: Is there evidence that the result was not biased by missing outcome data?		x				
	3.3 If N/PN to 3.2: Could missingness in the outcome depend on its true value?					x	
	3.4 If Y/PY/NI to 3.3: Is it likely that missingness in the outcome depended on its true value?					x	
	Risk-of-bias judgment (**low/**high/some concerns)						

RoB2	for Rudzianskas et al. 2014	Y	PY	N	PN	NI	NA
	**4. Bias in measurement of the outcome**						
	4.1 Was the method of measuring the outcome inappropriate?					x	
	4.2 Could measurement or ascertainment of the outcome have differed between intervention groups?					x	
	4.3 If N/PN/NI to 4.1 and 4.2: Were outcome assessors aware of the intervention received by study participants?	x					
	4.4 If Y/PY/NI to 4.3: Could assessment of the outcome have been influenced by knowledge of intervention received?		x				
	4.5 If Y/PY/NI to 4.4: Is it likely that assessment of the outcome was influenced by knowledge of intervention received?		x				
	Risk-of-bias judgment (low/**high**/some concerns)						
	**5. Bias in selection of the reported results**						
	5.1 Were the data that produced this result analysed in accordance with a prespecified analysis plan that was finalised before unblinded outcome data were available for analysis?					x	
	Is the numerical result being assessed likely to have been selected, on the basis of the results, from:						
	5.2 ... multiple eligible outcome measurements (eg, scales, definitions, time points) within the outcome domain?	x					
	5.3 ... multiple eligible analyses of the data?	x					
	Risk-of-bias judgment (low/high/**some concerns**)						
	**Overall bias**						
	Risk-of-bias judgment (low/high/**some concerns**)						

Supplementary Material (I): Overall Incidence of ORN

Study	Events	Total	Proportion	95%-CI	Weight (common)	Weight (random)
Al_Wassia et al., 2015	5	27	0.19	[0.06; 0.38]	3.3%	2.7%
Awan et al., 2018	0	45	0.00	[0.00; 0.08]	0.4%	0.8%
Bhalavat et al., 2018	0	14	0.00	[0.00; 0.23]	0.4%	0.8%
Bahl et al., 2018	0	20	0.00	[0.00; 0.17]	0.4%	0.8%
Beddok et al., 2023	5	23	0.22	[0.07; 0.44]	3.1%	2.7%
Benchalal et al., 1995	2	19	0.11	[0.01; 0.33]	1.4%	1.9%
Berger et al., 2010	0	57	0.00	[0.00; 0.06]	0.4%	0.8%
Bots et al., 2017	8	137	0.06	[0.03; 0.11]	6.1%	3.2%
Boustani et al., 2021	6	33	0.18	[0.07; 0.35]	3.9%	2.9%
Buglione et al., 2015	1	75	0.01	[0.00; 0.07]	0.8%	1.3%
Bussu et al., 2024	0	34	0.00	[0.00; 0.10]	0.4%	0.8%
Cengiz et al., 2011	1	46	0.02	[0.00; 0.12]	0.8%	1.3%
Chen et al., 2007	0	113	0.00	[0.00; 0.03]	0.4%	0.8%
Chen et al., 2011	0	21	0.00	[0.00; 0.16]	0.4%	0.8%
Crevoisier et al., 2001	4	25	0.16	[0.05; 0.36]	2.7%	2.5%
Cvek et al., 2016	4	40	0.10	[0.03; 0.24]	2.9%	2.6%
Dionisi et al., 2019	1	17	0.06	[0.00; 0.29]	0.8%	1.3%
Dornoff et al., 2015	0	66	0.00	[0.00; 0.05]	0.4%	0.8%
Duprez et al., 2009	3	84	0.04	[0.01; 0.10]	2.3%	2.4%
Embring et al., 2021	4	54	0.07	[0.02; 0.18]	3.0%	2.6%
Hall et al., 2003	2	18	0.11	[0.01; 0.35]	1.4%	1.9%
Hegde et al., 2018	1	20	0.05	[0.00; 0.25]	0.8%	1.3%
Hepel et al., 2005	1	30	0.03	[0.00; 0.17]	0.8%	1.3%
Held et al., 2019	3	229	0.01	[0.00; 0.04]	2.4%	2.4%
Heron et al., 2009	0	25	0.00	[0.00; 0.14]	0.4%	0.8%
Hsieh et al., 2024	4	22	0.18	[0.05; 0.40]	2.6%	2.5%
Hoell et al., 2023	1	45	0.02	[0.00; 0.12]	0.8%	1.3%
Hwang et al., 1998	2	64	0.03	[0.00; 0.11]	1.6%	2.0%
Jiang et al., 2019	0	61	0.00	[0.00; 0.06]	0.4%	0.8%
Jensen et al., 2010	0	21	0.00	[0.00; 0.16]	0.4%	0.8%
Kakria et al., 2014	0	31	0.00	[0.00; 0.11]	0.4%	0.8%
Karam et al., 2016	1	42	0.02	[0.00; 0.13]	0.8%	1.3%
Kramer et al., 2005	2	38	0.05	[0.01; 0.18]	1.5%	1.9%
Lee et al., 2017	1	20	0.05	[0.00; 0.25]	0.8%	1.3%
Lee et al., 2016	2	66	0.03	[0.00; 0.11]	1.6%	2.0%
Low et al., 2006	6	36	0.17	[0.06; 0.33]	4.0%	2.9%
Megahed et al., 2024	0	9	0.00	[0.00; 0.34]	0.4%	0.8%
Martínez_Fernández e, 2017	3	63	0.05	[0.01; 0.13]	2.3%	2.4%
Orlandi et al., 2019	2	159	0.01	[0.00; 0.04]	1.6%	2.0%
Ozyigit et al., 2011	0	27	0.00	[0.00; 0.13]	0.4%	0.8%
Pialat et al., 2021	1	25	0.04	[0.00; 0.20]	0.8%	1.3%
Ritter et al., 2016	0	15	0.00	[0.00; 0.22]	0.4%	0.8%
Roh et al., 2009	1	36	0.03	[0.00; 0.15]	0.8%	1.3%
Romesser et al., 2016	1	92	0.01	[0.00; 0.06]	0.8%	1.3%
Rühle et al., 2020	2	48	0.04	[0.01; 0.14]	1.5%	2.0%
Rudzianskas et al., 2014	3	63	0.05	[0.01; 0.13]	2.3%	2.4%
Schaefer et al., 2000	0	32	0.00	[0.00; 0.11]	0.4%	0.8%
Salama et al., 2006	13	115	0.11	[0.06; 0.19]	9.3%	3.5%
Seiwert et al., 2008	3	29	0.10	[0.02; 0.27]	2.2%	2.3%
Siddiqui et al., 2009	1	21	0.05	[0.00; 0.24]	0.8%	1.3%
Spencer et al., 2007	0	79	0.00	[0.00; 0.05]	0.4%	0.8%
Suh et al., 2008	1	12	0.08	[0.00; 0.38]	0.7%	1.2%
Sulman et al., 2009	4	74	0.05	[0.01; 0.13]	3.0%	2.6%
Takiar et al., 2016	14	206	0.07	[0.04; 0.11]	10.5%	3.5%
Teo et al., 1998	2	117	0.02	[0.00; 0.06]	1.6%	2.0%
Tselis et al., 2011	2	74	0.03	[0.00; 0.09]	1.6%	2.0%
Uysal et al., 2023	0	10	0.00	[0.00; 0.31]	0.4%	0.8%
Vischioni et al., 2020	1	51	0.02	[0.00; 0.10]	0.8%	1.3%
Voynov et al., 2006	0	22	0.00	[0.00; 0.15]	0.4%	0.8%
Waldron et al., 2019	1	26	0.04	[0.00; 0.20]	0.8%	1.3%
Zheng et al., 2004	1	117	0.01	[0.00; 0.05]	0.8%	1.3%
Zwicker et al., 2011	0	38	0.00	[0.00; 0.09]	0.4%	0.8%
**Common effect model**		**3278**	**0.06**	**[0.05; 0.07]**	**100.0%**	**.**
**Random effects model**			**0.05**	**[0.04; 0.06]**	**.**	**100.0%**

Heterogeneity: $I^2 = 42.4\%$, $\tau^2 = 0.4768$, $\chi^2_{61} = 105.97$ ($p = 0.0003$)

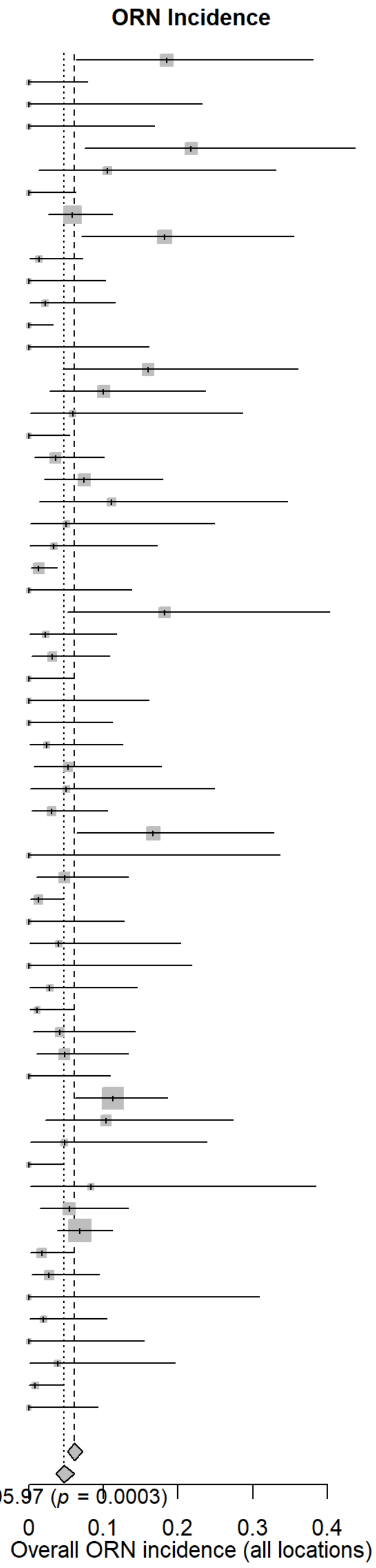

Supplementary Material (I): Incidence of ORN of the Mandible

Study	Events	Total	Proportion	95% CI	Weight (common)	Weight (random)
Al_Wassia et al., 2015	5	27	0.19	[0.06; 0.38]	4.7%	3.2%
Awan et al., 2018	0	45	0.00	[0.00; 0.08]	0.6%	1.0%
Bhalavat et al., 2018	0	14	0.00	[0.00; 0.23]	0.6%	1.0%
Bahl et al., 2018	0	20	0.00	[0.00; 0.17]	0.6%	1.0%
Beddok et al., 2023	5	23	0.22	[0.07; 0.44]	4.5%	3.2%
Benchalal et al., 1995	1	19	0.05	[0.00; 0.26]	1.1%	1.6%
Berger et al., 2010	0	57	0.00	[0.00; 0.06]	0.6%	1.0%
Bots et al., 2017	5	137	0.04	[0.01; 0.08]	5.6%	3.4%
Boustani et al., 2021	0	33	0.00	[0.00; 0.11]	0.6%	1.0%
Buglione et al., 2015	1	75	0.01	[0.00; 0.07]	1.1%	1.6%
Bussu et al., 2024	0	34	0.00	[0.00; 0.10]	0.6%	1.0%
Cengiz et al., 2011	1	46	0.02	[0.00; 0.12]	1.1%	1.6%
Chen et al., 2007	0	113	0.00	[0.00; 0.03]	0.6%	1.0%
Chen et al., 2011	0	21	0.00	[0.00; 0.16]	0.6%	1.0%
Crevoisier et al., 2001	4	25	0.16	[0.05; 0.36]	3.9%	3.0%
Cvek et al., 2016	4	40	0.10	[0.03; 0.24]	4.1%	3.1%
Dionisi et al., 2019	0	17	0.00	[0.00; 0.20]	0.6%	1.0%
Dornoff et al., 2015	0	66	0.00	[0.00; 0.05]	0.6%	1.0%
Duprez et al., 2009	1	84	0.01	[0.00; 0.06]	1.1%	1.6%
Embring et al., 2021	1	54	0.02	[0.00; 0.10]	1.1%	1.6%
Hall et al., 2003	0	18	0.00	[0.00; 0.19]	0.6%	1.0%
Hegde et al., 2018	1	20	0.05	[0.00; 0.25]	1.1%	1.6%
Hepel et al., 2005	1	30	0.03	[0.00; 0.17]	1.1%	1.6%
Heron et al., 2009	0	25	0.00	[0.00; 0.14]	0.6%	1.0%
Hsieh et al., 2024	4	22	0.18	[0.05; 0.40]	3.8%	3.0%
Hoell et al., 2023	1	45	0.02	[0.00; 0.12]	1.1%	1.6%
Hwang et al., 1998	0	64	0.00	[0.00; 0.06]	0.6%	1.0%
Jiang et al., 2019	0	61	0.00	[0.00; 0.06]	0.6%	1.0%
Jensen et al., 2010	0	21	0.00	[0.00; 0.16]	0.6%	1.0%
Kakria et al., 2014	0	31	0.00	[0.00; 0.11]	0.6%	1.0%
Karam et al., 2016	0	42	0.00	[0.00; 0.08]	0.6%	1.0%
Kramer et al., 2005	2	38	0.05	[0.01; 0.18]	2.2%	2.4%
Lee et al., 2017	0	20	0.00	[0.00; 0.17]	0.6%	1.0%
Low et al., 2006	0	36	0.00	[0.00; 0.10]	0.6%	1.0%
Megahed et al., 2024	0	9	0.00	[0.00; 0.34]	0.5%	1.0%
Martínez_Fernández et al., 2017	3	63	0.05	[0.01; 0.13]	3.3%	2.9%
Orlandi et al., 2019	2	159	0.01	[0.00; 0.04]	2.3%	2.4%
Ozyigit et al., 2011	0	27	0.00	[0.00; 0.13]	0.6%	1.0%
Pialat et al., 2021	0	25	0.00	[0.00; 0.14]	0.6%	1.0%
Ritter et al., 2016	0	15	0.00	[0.00; 0.22]	0.6%	1.0%
Roh et al., 2009	1	36	0.03	[0.00; 0.15]	1.1%	1.6%
Romesser et al., 2016	1	92	0.01	[0.00; 0.06]	1.1%	1.6%
Rühle et al., 2020	2	48	0.04	[0.01; 0.14]	2.2%	2.4%
Rudzianskas et al., 2014	3	63	0.05	[0.01; 0.13]	3.3%	2.9%
Schaefer et al., 2000	0	32	0.00	[0.00; 0.11]	0.6%	1.0%
Salama et al., 2006	13	115	0.11	[0.06; 0.19]	13.3%	4.1%
Seiwert et al., 2008	3	29	0.10	[0.02; 0.27]	3.1%	2.8%
Siddiqui et al., 2009	1	21	0.05	[0.00; 0.24]	1.1%	1.6%
Spencer et al., 2007	0	79	0.00	[0.00; 0.05]	0.6%	1.0%
Suh et al., 2008	1	12	0.08	[0.00; 0.38]	1.1%	1.6%
Sulman et al., 2009	3	74	0.04	[0.01; 0.11]	3.3%	2.9%
Takiar et al., 2016	5	206	0.02	[0.01; 0.06]	5.6%	3.4%
Teo et al., 1998	1	117	0.01	[0.00; 0.05]	1.1%	1.6%
Tselis et al., 2011	1	74	0.01	[0.00; 0.07]	1.1%	1.6%
Uysal et al., 2023	0	10	0.00	[0.00; 0.31]	0.6%	1.0%
Vischioni et al., 2020	1	51	0.02	[0.00; 0.10]	1.1%	1.6%
Voynov et al., 2006	0	22	0.00	[0.00; 0.15]	0.6%	1.0%
Waldron et al., 2019	1	26	0.04	[0.00; 0.20]	1.1%	1.6%
Zheng et al., 2004	1	117	0.01	[0.00; 0.05]	1.1%	1.6%
Zwicker et al., 2011	0	38	0.00	[0.00; 0.09]	0.6%	1.0%
**Common effect model**		**2983**	**0.05**	**[0.04; 0.06]**	**100.0%**	.
**Random effects model**			**0.04**	**[0.03; 0.05]**	.	**100.0%**
**Prediction interval**				**[0.01; 0.14]**		

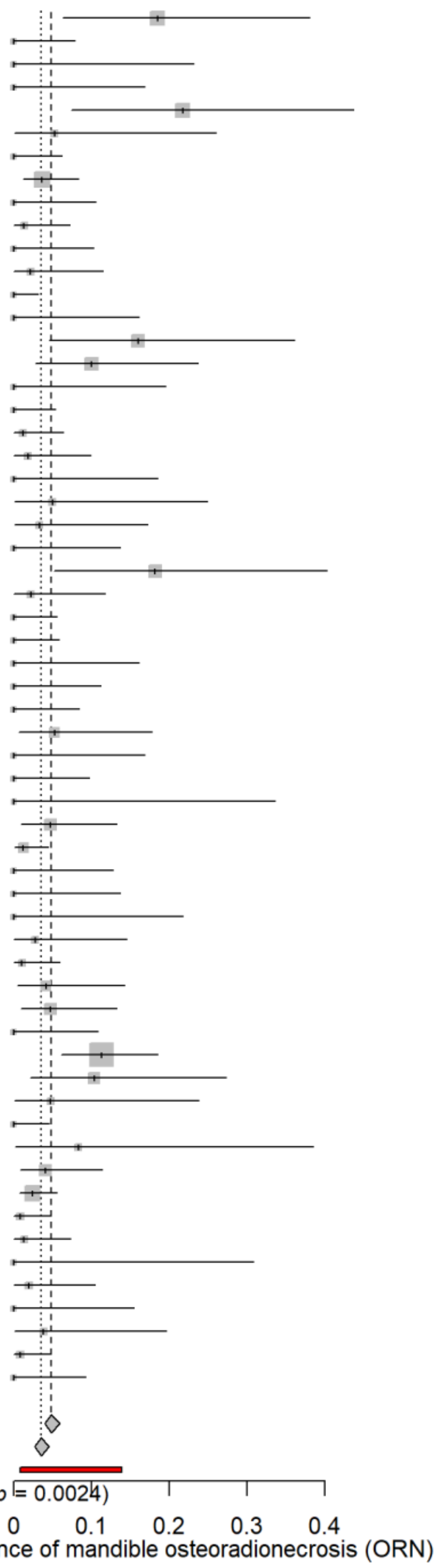

Heterogeneity: $I^2 = 37.4\%$, $\tau^2 = 0.5355$, $\chi^2_{59} = 94.20$ ($p = 0.0024$)
