# Supplementary material for: Osteoradionecrosis in patients with head and neck cancer after reirradiation – a systematic review and meta-analysis
Source: Clin Transl Radiat Oncol. 2026 Oct 28;62:101342. doi: 10.1016/j.ctro.2026.101342 (PMC13744302; doi:10.1016/j.ctro.2026.101342)
Supplement: Supplementary file 2 [file mmc3.pdf]

Supplementary Methods: Statistical Analysis

Data management and preprocessing were conducted using the packages *readxl*, *tidyverse*, *dplyr*, *stringr*, and *tibble*, while meta-analyses and meta regressions were performed using the *meta* and *metafor* packages. Automated reporting and text generation were supported using the *glue* package. Pooled estimates were calculated using a random-effects model to account for between-study heterogeneity.

Publication bias and small-study effects were assessed using funnel plot visualization and Egger's regression test. Funnel plots were constructed by plotting study-specific logit-transformed ORN proportions against their standard errors, with the pooled random-effects estimate used as the reference line. Egger's test was based on a weighted linear regression of the standard normal deviate on the standard error.[1]

Proportions were transformed using a logit transformation prior to pooling to stabilize variances. The pooled cumulative incidence and corresponding 95% confidence intervals (CIs) were then calculated and back-transformed for reporting. Statistical heterogeneity between studies was assessed using the I^2 statistic and Cochran's Q test. I^2 values of 25%, 50%, and 75% were considered indicative of low, moderate, and high heterogeneity, respectively. For the primary analysis, 95% CIs and prediction intervals were calculated using the Hartung–Knapp method; for the secondary analyses (patients with available follow-up and ORN at all anatomical sites), 95% CIs were based on the normal distribution and prediction intervals on a t-distribution with $k-1$ degrees of freedom. In all cases, τ^2 was estimated by restricted maximum likelihood and intervals were back-transformed from the logit scale.

As individual patient data were not available, continuous variables reported as medians in the included studies (e.g., age, radiation dose, follow-up duration, and interval between radiotherapy courses) were summarized as pooled median by calculating the median of the reported study medians. These values therefore represent study-level summary measures rather than true patient-level medians. To account for methodological quality, study-specific scores derived from the MINORS and JBI assessments were converted into percentage values and incorporated into the meta-analysis as an additional weighting factor. Where sufficient data was available, subgroup analyses were performed to explore potential sources of heterogeneity. A two-sided p-value <.05 was considered statistically significant.

Reference:

[1] Egger M, Davey Smith G, Schneider M, Minder C. Bias in meta-analysis detected by a simple, graphical test. BMJ. 1997;315:629-34.
